# Longitudinal assessment of skeletal muscle functional mechanics in the DE50-MD dog model of Duchenne muscular dystrophy

**DOI:** 10.1242/dmm.050395

**Published:** 2023-12-20

**Authors:** Dominique O. Riddell, John C. W. Hildyard, Rachel C. M. Harron, Frances Taylor-Brown, Joe N. Kornegay, Dominic J. Wells, Richard J. Piercy

**Affiliations:** ^1^ Comparative Neuromuscular Diseases Laboratory, Department of Clinical Science and Services, Royal Veterinary College, London NW10TU, UK; ^2^ Texas A&M University, College of Veterinary Medicine and Biomedical Sciences, College Station, TX 77843, USA; ^3^ Department of Comparative Biomedical Sciences, Royal Veterinary College, London NW10TU, UK

**Keywords:** Physiology, Force, Eccentric exercise, Dog, DE50-MD, Duchenne muscular dystrophy

## Abstract

Duchenne muscular dystrophy (DMD), caused by mutations in the dystrophin (*DMD*) gene, is associated with fatal muscle degeneration and atrophy. Patients with DMD have progressive reductions in skeletal muscle strength and resistance to eccentric muscle stretch. Using the DE50-MD dog model of DMD, we assessed tibiotarsal joint (TTJ) flexor and extensor force dynamics, and the resistance of dystrophic muscle to eccentric stretch. Male DE50-MD and wild-type (WT) dogs were analysed every 3 months until 18 months of age. There was an age-associated decline in eccentric contraction resistance in DE50-MD TTJ flexors that discriminated, with high statistical power, WT from DE50-MD individuals. For isometric contraction, at the majority of timepoints, DE50-MD dogs had lower maximum absolute and relative TTJ flexor force, reduced TTJ muscle contraction times and prolonged relaxation compared to those in WT dogs. Cranial tibial muscles, the primary TTJ flexor, of 18-month-old DE50-MD dogs had significant numbers of regenerating fibres as expected, but also fewer type I fibres and more hybrid fibres than those in WT dogs. We conclude that these parameters, in particular, the eccentric contraction decrement, could be used as objective outcome measures for pre-clinical assessment in DE50-MD dogs.

## Teaser

**Summary:** The DE50-MD dog model of Duchenne muscular dystrophy shows a marked age-associated torque reduction following eccentric exercise; this biomarker is a strong candidate for objective measurement of muscle function for preclinical trials.

## INTRODUCTION

Duchenne muscular dystrophy (DMD), a fatal, X-linked, muscle-wasting disease with no cure, is caused by mutations in the dystrophin (*DMD*) gene. There is an urgent need for effective treatments, but these require testing in clinically relevant animal disease models. Although DMD mouse models are useful for assessment of biochemical efficacy of therapies, they fail to display an overt phenotype; in contrast, dog models are closer in size to humans and their clinically relevant phenotypes makes them especially well-suited to demonstration of functional efficacy of novel treatments (Wells, 2018).

Improvement in muscle function is a critical measure of success for therapeutic trials; however, objectively measuring it can be challenging in both patients with DMD and in animal models. In humans, standardised tests measure the ability (or time required) to perform certain tasks (Bushby et al., 2010). Commonly used tests include the 6-min walk test (McDonald et al., 2010) and the North Star ambulatory assessment (Scott et al., 2012). Such tests rely on competence and consistency of an evaluator, and compliance of the patient (Nagy et al., 2019); the latter element in particular also applies to conscious muscle function tests in dogs. The 6-min walk test has been adapted for assessment of muscle function and endurance in dogs (Acosta et al., 2016), but these studies revealed that the distance walked varies greatly between affected animals and correlates poorly with other phenotypic biomarkers. Objective measures of muscle function are therefore preferable as key biomarkers in trials.

In the anaesthetised *mdx* mouse, both *in vivo*- and *ex vivo-*established objective protocols exist for measuring pelvic limb muscle force: *in vivo* quantification of muscle torque can be achieved by positioning the paw on a force-transducing pedal, with percutaneous needle electrode stimulation of the nerves supplying the flexor muscles of the tibiotarsal (ankle) joint (TTJ) (Capogrosso et al., 2017). This *in vivo* protocol has been adapted and used in anaesthetised dystrophic dog models (Childers et al., 2011). *In situ* protocols have also been established for measurement of muscle force and kinetics for the pelvic limb muscles of mice (Wells, 2019) and for the extensor carpi ulnaris muscle of dogs (Hakim et al., 2021); in these terminal protocols (conducted in anaesthetised animals), the distal tendon of the muscle is cut and directly attached to a lever arm/position controller while maintaining intact nerve and blood supplies. An additional *in vivo* protocol has been established for assessment of the peroneus longus muscle in dogs by a tendon-suture method that allows longitudinal studies (Kornegay et al., 1994). Terminal *ex vivo* protocols also exist for mouse models to quantify the force-generating capacity of individual muscles, including the extensor digitorum longus, soleus and diaphragm muscles, which are small or thin enough to allow sufficient *ex vivo* perfusion of oxygen and removal of waste metabolites (Moorwood et al., 2013; Wells, 2019).

Following supramaximal stimulation, the pelvic limb muscles of *mdx* mice typically generate greater absolute force during isometric contraction compared to that of age-matched controls (https://www.treat-nmd.org/wp-content/uploads/2023/07/DMD_M.1.2.002.pdf). This greater absolute force reflects the compensatory hypertrophy that occurs in the limb muscles of *mdx* mice, a feature that does not occur in the majority of muscles in patients with DMD (Stedman et al., 1991). When these results are normalised to the cross-sectional area (to estimate specific force), *mdx* muscles have a lower specific force than that of wild-type (WT) muscle (https://www.treat-nmd.org/wp-content/uploads/2023/07/DMD_M.1.2.002.pdf). The mechanism causing weakness of dystrophic muscle is generally explained by a decrease in active contractile apparatus due to muscle fibre necrosis and fibrosis, with fat and inflammatory cell infiltration (Garlich et al., 2010; Blake et al., 2002). Several studies have also demonstrated reduced action potential-induced calcium ion release from the sarcoplasmic reticulum in dystrophic muscle, which might contribute to lower force generation compared to that of healthy muscle (Ludin, 1973; Woods et al., 2004; Hollingworth et al., 2008; Head, 2010; Miles et al., 2011; García-Castañeda et al., 2022).

In contrast to the *mdx* mouse, DMD dog models typically exhibit marked muscle atrophy (Kornegay et al., 2012a; Hornby et al., 2021) and lower absolute and specific isometric muscle forces (Kornegay et al., 1994, 1999; Hakim et al., 2021) compared to those of healthy controls. Unlike terminal muscle physiology studies that allow removal of the stimulated muscle for accurate quantification of cross-sectional area, calculation of specific force for longitudinal, *in vivo* canine studies generally uses bodyweight as a normalisation factor (Kornegay et al., 1999). As is the case with the *mdx* mouse model and patients with DMD, there is marked phenotypic variation across dystrophic canine muscles (Kornegay et al., 2012b). Notably, atrophy is not consistent: the cranial sartorius muscle hypertrophies in the Golden Retriever muscular dystrophy (GRMD) dog model (Kornegay et al., 2003) and shows no difference in muscle volume between genotypes in the DE50-MD dog model (Hornby et al., 2021). Muscle force in dystrophic dogs is also dependent on age, exemplified in the GRMD dog model in which marked changes occur over the first year of life, with TTJ flexor force being significantly lower at 3 months of age, whereas the TTJ extensor muscles show the greatest reductions in force at later ages (Kornegay et al., 1999).

Muscle performance can also be evaluated by measuring response to eccentric contractions (ECs), where the muscle lengthens while contracting (typically to generate a braking force, which is higher than that exerted by isometric contractions) (Ingalls et al., 1998). ECs can be induced electrophysiologically by exerting mechanical stretch on muscles under tetanic contraction. Compared to WT controls, dystrophic muscle in both the *mdx* mouse and the GRMD dog is more susceptible to injury from ECs and has greater force loss with repetitive contractions (Moens et al., 1993; Petrof et al., 1993; Dellorusso et al., 2001; Childers et al., 2002; Tegeler et al., 2010). The mechanism underlying the susceptibility of dystrophic muscle to ECs is not fully understood, but has been attributed to damage of the muscle contractile unit structure (Koh and Escobedo, 2004; Blaauw et al., 2010; Call et al., 2011), neuromuscular junction disruption (Pratt et al., 2013, 2015), defects of excitation-contraction coupling (Warren et al., 2001) and decreased muscle excitability (Call et al., 2013; Roy et al., 2016). In the *mdx* mouse, eccentric stretch protocols using the tibialis anterior muscle (which flexes the TTJ) demonstrate marked percentage force loss immediately following ECs; the loss in force becomes greater with age (conversely, age-matched healthy muscle typically shows little or no force loss) (Dellorusso et al., 2001). Work investigating similar age-associated increases in eccentric stretch susceptibility in dystrophic dog models is more limited. Whereas WT dog muscle showed improved resistance to eccentric stretch at 6 versus 3 months of age, GRMD dogs showed a significant decline in resistance between 3 and 6 months (Childers et al., 2012; Kornegay et al., 2014). In both GRMD and *mdx* models, EC protocol results better differentiate affected from age-matched controls than measurement of isometric force (Dellorusso et al., 2001).

The GRMD dog is the classic canine model of DMD and has commonly been used to bridge the gap between mouse models and trials in human patients (Kornegay, 2017). These animals carry a splice site mutation within intron 6, leading to loss of exon 7 from the mature dystrophin mRNA. In contrast, the DE50-MD dog is a newly established canine model of DMD that has a mutation in a mutational hotspot (exons 44-53) in the *DMD* gene: DE50-MD dogs harbour a point mutation in the donor splice site of intron 50 that results in deletion of exon 50 from *DMD* gene transcripts (Walmsley et al., 2010). To characterise the phenotype of the DE50-MD model, we conducted an extensive natural history study, monitoring disease progression longitudinally over 18 months via multiple complementary approaches (including the muscle physiology measurements described here). This work recently concluded, and results of other investigations suggest that the model exhibits disease progression comparable to that seen in young human patients with DMD: covering the period from first symptomatic manifestation (at approximately 3 years of age) to before loss of ambulation (typically between 10 and 12 years of age) (Hildyard et al., 2018a; Riddell et al., 2021, 2022; Hornby et al., 2021). Although magnetic resonance imaging (MRI) measurements of DE50-MD dogs muscle volumes did not differ from WT dogs at 3 months of age, the volumes were significantly smaller in DE50-MD dogs at 6 months of age and onwards (except for the cranial sartorius, as discussed above; Hornby et al., 2021). Furthermore, histological evaluation of skeletal muscle reveals that DE50-MD muscle exhibits profound inflammatory changes and progressive fibrosis (Hildyard et al., 2022).

In this study, we present the results of our longitudinal assessment of isometric contraction force and contraction/relaxation dynamics in DE50-MD and WT dogs, and the effects of age on susceptibility of DE50-MD muscle to EC-induced torque loss. A principal goal was to establish (via power/sample size calculations) muscle physiology outcome measures that are suitable as biomarkers, allowing future studies evaluating the functional efficacy of treatment to be conducted with statistically rigorous experimental design, a critical prerequisite for ethical animal use.

## RESULTS

### Isometric contraction

**Fig. 1.**
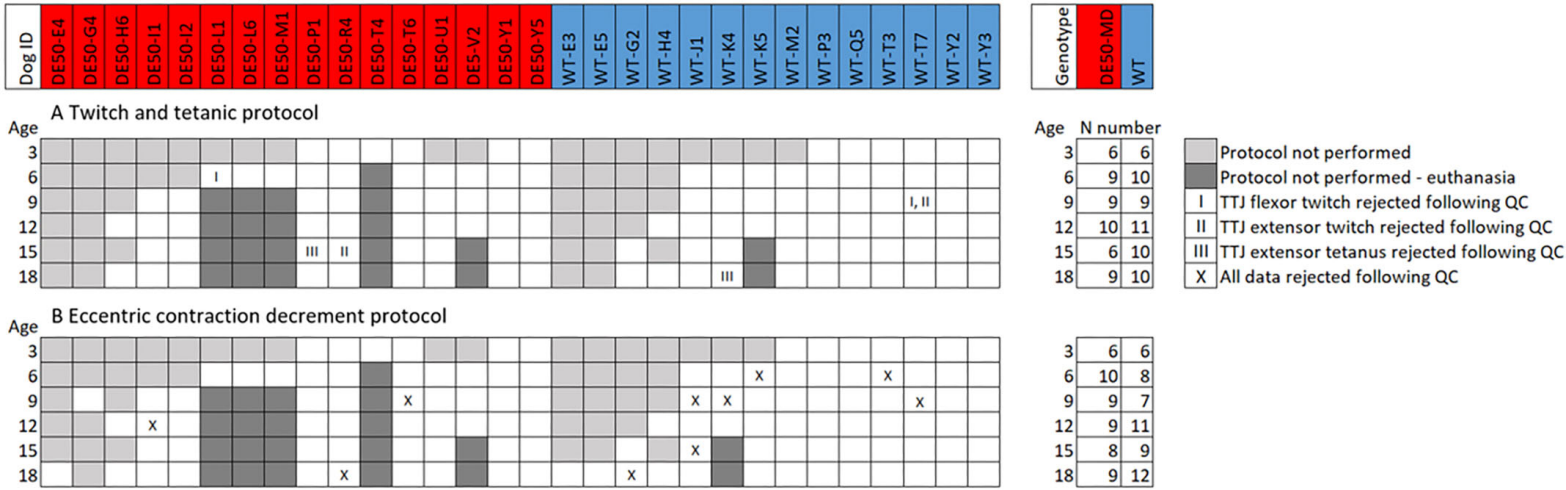
**Muscle physiology sampling timepoints for each study dog.** Data collected and analysed for (A) twitch and tetanic protocols and (B) eccentric contraction (EC) decrement (ECD) protocol. Age is displayed in months. Dog ID shows the study name of each individual animal included in this study: dog IDs with the prefix ‘DE50’ are DE50-MD dogs (red), dogs with the prefix ‘WT’ are wild-type dogs (blue). White boxes show timepoints for which data were collected and analysed. Grey boxes indicate timepoints for which data were not collected: dark grey boxes indicate timepoints that were not tested due to animal euthanasia prior to this timepoint, whereas light grey boxes indicate that data were not collected for other reasons. In some cases, the collected data were rejected based on quality control (QC) checks: in the case of twitch and tetanic data, this was necessary for two tibiotarsal joint (TTJ) flexor twitches (I), two TTJ extensor twitches (II) and two TTJ extensor tetani (III), and all data were rejected from ten ECD protocol readings (X) (see ‘Eccentric contraction protocol data’ at https://doi.org/10.6084/m9.figshare.23501319 for more details and raw data).

In total, data from 16 DE50-MD and 14 WT dogs are included in this study (Fig. 1). Dogs were studied at 3-monthly intervals, from 3 to 18 months of age. Missing data occurred for several reasons including early euthanasia due to reaching pre-determined humane endpoints relating to the disease phenotype (dysphagia) or due to unrelated health conditions (see Materials and Methods). Muscle torque was evaluated for the tibiotarsal flexor and extensor muscle groups, and was performed using an Aurora Scientific large animal frame with a force-transducing foot pedal and dual-mode lever system (Fig. S1). Muscle torque procedures were conducted with the dog under general anaesthesia in dorsal recumbency. The tibiotarsal (ankle), stifle (knee) and coxofemoral (hip) joints were positioned at 90° angles and the tarsus was taped with bandaging wrap to the force transducing foot-pedal. For twitch and tetanus isometric torque, the tibial and fibular nerves were individually supramaximally stimulated via percutaneous electrodes. For each twitch and tetanus, the following parameters were measured: maximum force of contraction, tetanus:torque ratio, time to contraction and time to relaxation (Fig. S2).

#### Maximum force of contraction

**Fig. 2.**
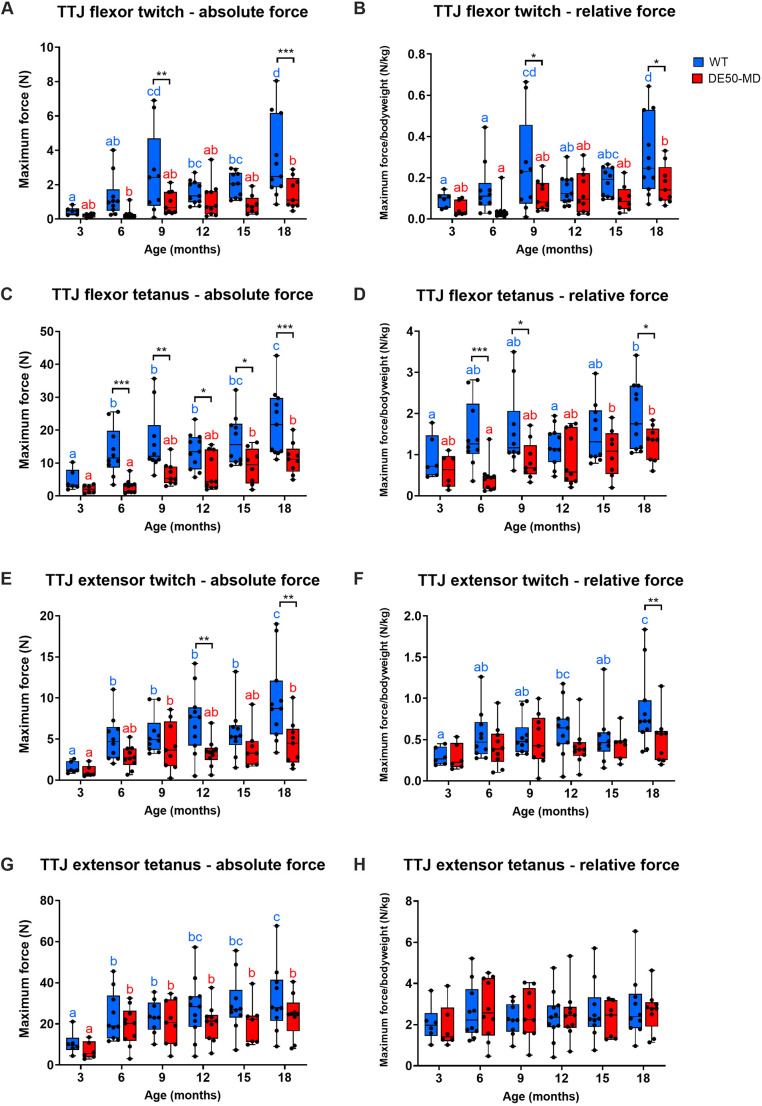
**Absolute and relative maximum force of contraction for TTJ flexion and extension following fibular and tibial nerve twitch and tetanus.** Results for dogs aged between 3 and 18 months showing: TTJ flexor twitch absolute force (A) and relative force (B); TTJ flexor tetanus absolute force (C) and relative force (D); TTJ extensor twitch absolute force (E) and relative force (F); TTJ extensor tetanus absolute force (G) and relative force (F). Absolute force is shown in Newtons (N) and relative force in N/kg. Boxes extend from the 25th to 75th percentile, with the lines within the boxes at the median value. Each point represents an individual DE50-MD (red; *n*=6-9) or WT (blue; *n*=5-11) dog, and whiskers show the minimum and maximum results for that age group. Asterisks denote the levels of significance of differences between the DE50-MD and WT genotypes based on linear mixed model and post hoc analysis with Holm–Šídák correction for multiple comparisons: *P<0.05; **P<0.01; ***P<0.001. Lowercase letters denote differences in the mean within the DE50-MD (red) or WT (blue) genotype; means sharing a letter are not significantly different.

DE50-MD muscles produced lower muscle force compared to that in WT muscles during TTJ flexor twitch [absolute force, *P*=0.0004; relative force (absolute force divided by bodyweight), *P*=0.002], TTJ flexor tetanus (absolute force, *P*<0.00001; relative force, *P*<0.00001) and TTJ extensor twitch (absolute force, *P*=0.002; relative force, *P*=0.046), with the greatest differences typically occurring at 6, 9 and 18 months of age (Fig. 2A-F; see Tables S1 and S2 for absolute and relative force data summary, respectively). The difference between genotypes was not significant for TTJ extensor tetanus force (absolute force, *P*=0.11; relative force, *P*=0.95; Fig. 2G-H). Absolute and relative force typically increased gradually between 3 and 18 months of age for both WT and DE50-MD dogs (age effect, *P*<0.05; Fig. 2A-G). An exception was relative TTJ extensor tetanus force, which showed no effect of age within either genotype (age effect, *P*=0.37, Fig. 2H).

#### Twitch:tetanus torque ratio

**Fig. 3.**
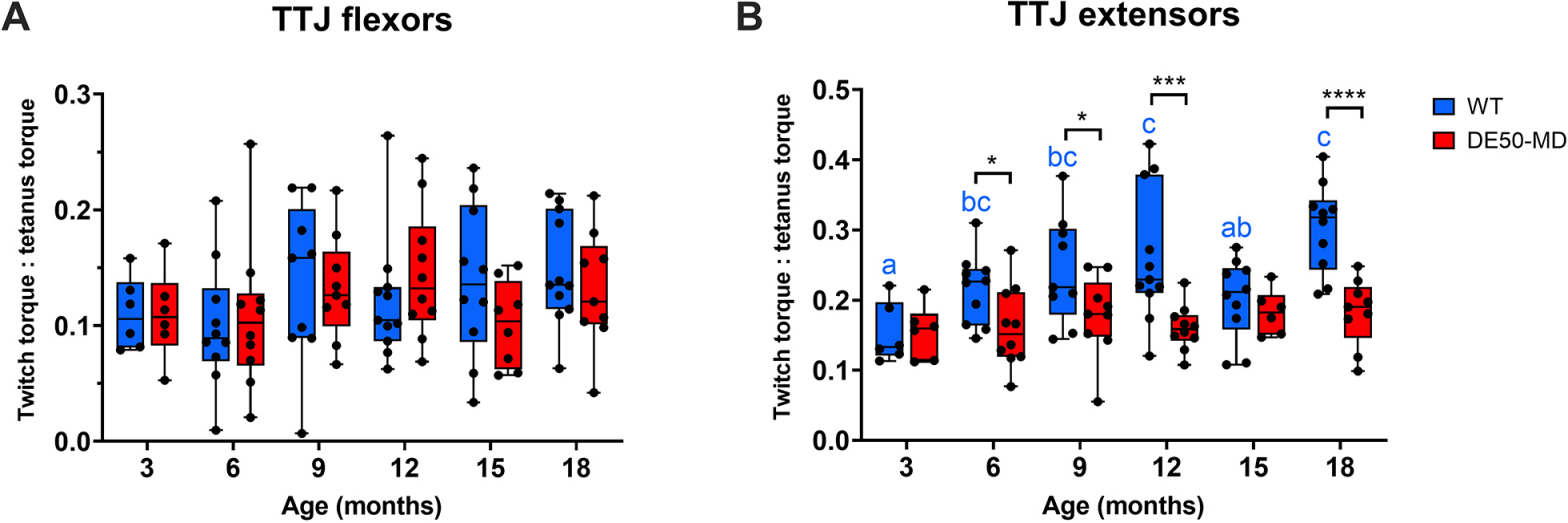
**Twitch to tetanus torque ratio.** The fraction of maximal tetanic torque generated by a single stimulus twitch, using the same needle electrode placement. Data are shown for stimulation of the TTJ flexors (A) and extensors (B). Needle electrodes were not moved between the twitch and tetanus stimulations. Results are shown for WT (blue; *n*=5-11) and DE50-MD (red; *n*=6-9) dogs studied longitudinally between 3 and 18 months of age. Asterisks denote the levels of significance of differences between the DE50-MD and WT genotypes based on linear mixed model and post hoc analysis with Holm–Šídák correction for multiple comparisons: *P<0.05; ***P<0.001; ****P<0.0001. Lowercase letters denote differences in the mean within the WT (blue) genotype; means sharing a letter are not significantly different.

The twitch:tetanus torque ratio (the fraction of maximal tetanic torque generated by a single stimulus twitch) was not significantly different between genotypes for the TTJ flexors (*P*=0.53; Fig. 3A), but was significantly higher in WT dogs for the TTJ extensors (*P*=0.0001; Fig. 3B). In addition, no effect of age (in either genotype) was observed with TTJ flexor stimulation (*P*=0.35), whereas TTJ extensor twitch:tetanus ratios showed a strong effect of age (*P*=0.003), increasing from 3 to 18 months in WT dogs, but not in DE50-MD dogs (see Table S3 for twitch:tetanus data summary).

#### Contraction dynamics

**Fig. 4.**
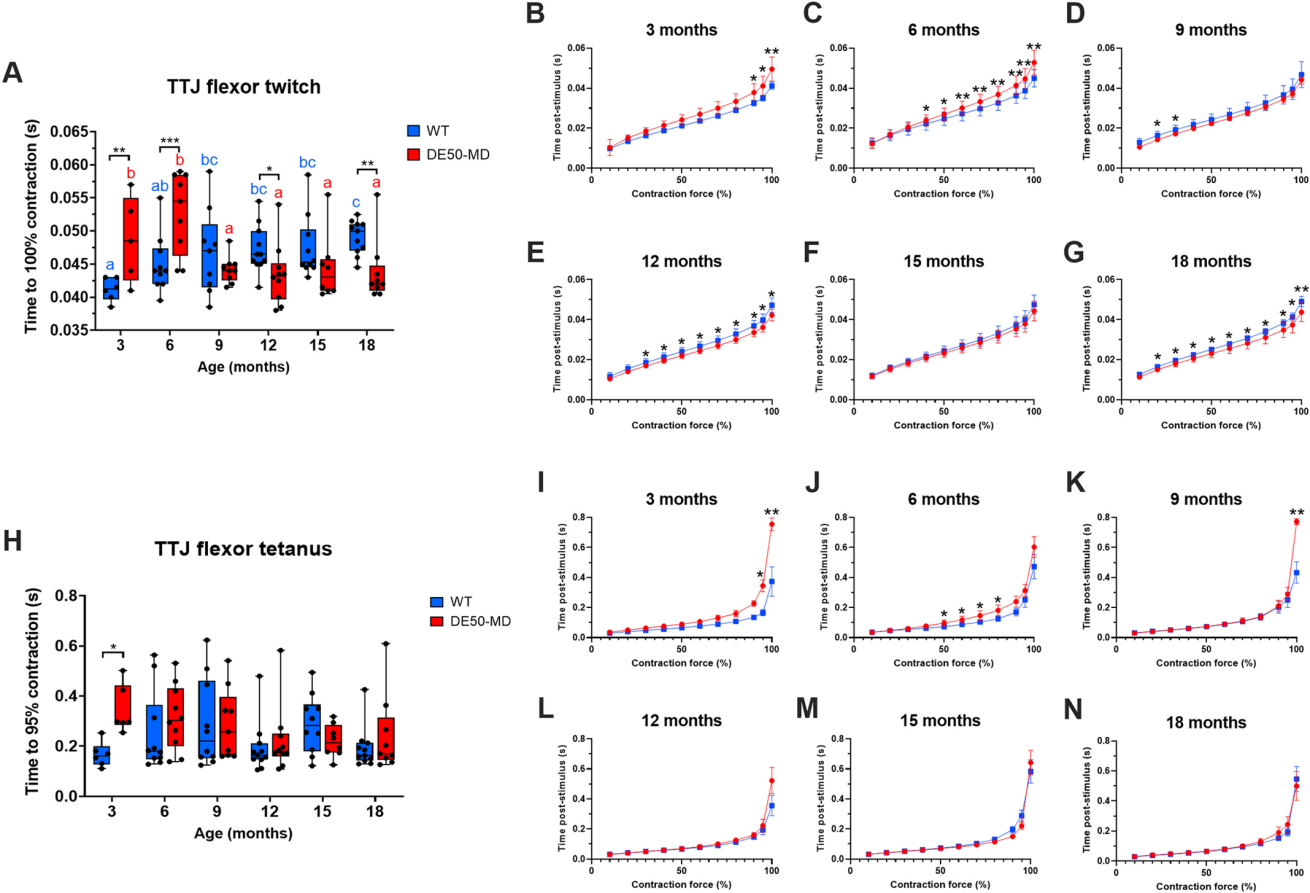
**Contraction time for TTJ flexors.** Comparison of WT (blue) and DE50-MD (red) dogs studied longitudinally from 3 to 18 months of age showing: (A) time to 100% TTJ flexor twitch contraction; (B-G) time course for contraction for TTJ flexor twitch in 10% increments; (H) time to 95% contraction for TTJ flexor tetanus; and (I-N) time course for contraction for TTJ flexor tetanus in 10% increments. For all graphs, *n*=5-11 WT dogs or 6-9 DE50-MD dogs. Asterisks denote the levels of significance of differences between DE50-MD and WT genotypes based on linear mixed model and post hoc analysis with Holm–Šídák correction for multiple comparisons: *P<0.05; **P<0.01; ***P<0.001. Letters denote differences in the mean within the DE50-MD (red) or WT (blue) genotype; means sharing a letter are not significantly different.

WT dogs took longer to reach maximum TTJ flexor twitch contraction at 12 and 18 months, whereas DE50-MD dogs took longer at 3 and 6 months of age (Fig. 4A, *P*<0.05). Analysis of contraction time in 10% increments showed that the TTJ flexor twitch of 3- and 6-month-old WT and DE50-MD dogs initially contracted at similar rates; however, DE50-MD dogs took longer to reach the later stages of contraction and the magnitude of this difference increased throughout the time course (Fig. 4B,C). From 9 to 18 months of age, both genotypes followed a more similar time course, with the TTJ flexor twitch of WT dogs contracting more slowly than that of 12- and 18-month-old DE50-MD dogs (Fig. 4D-G). In many cases, the plateau phase of the tetanic contractions showed a very gradual increase in force until the end of stimulation; however, this varied between individuals and within individuals of different ages – to account for this, the time taken to reach 95% contraction was compared rather than time taken to reach 100% contraction for tetanic contractions. As seen during TTJ flexor twitch, DE50-MD dogs took longer to reach 95% TTJ flexor tetanic force at 3 months of age than WT dogs (*P*=0.02, Fig. 4H), but no difference between genotypes was seen at any other timepoint. Comparison of the time course for contraction showed that the two genotypes closely overlapped at all ages (Fig. 4I-N), diverging significantly only in the second half of contraction in 3- to 9-month-old dogs.

**Fig. 5.**
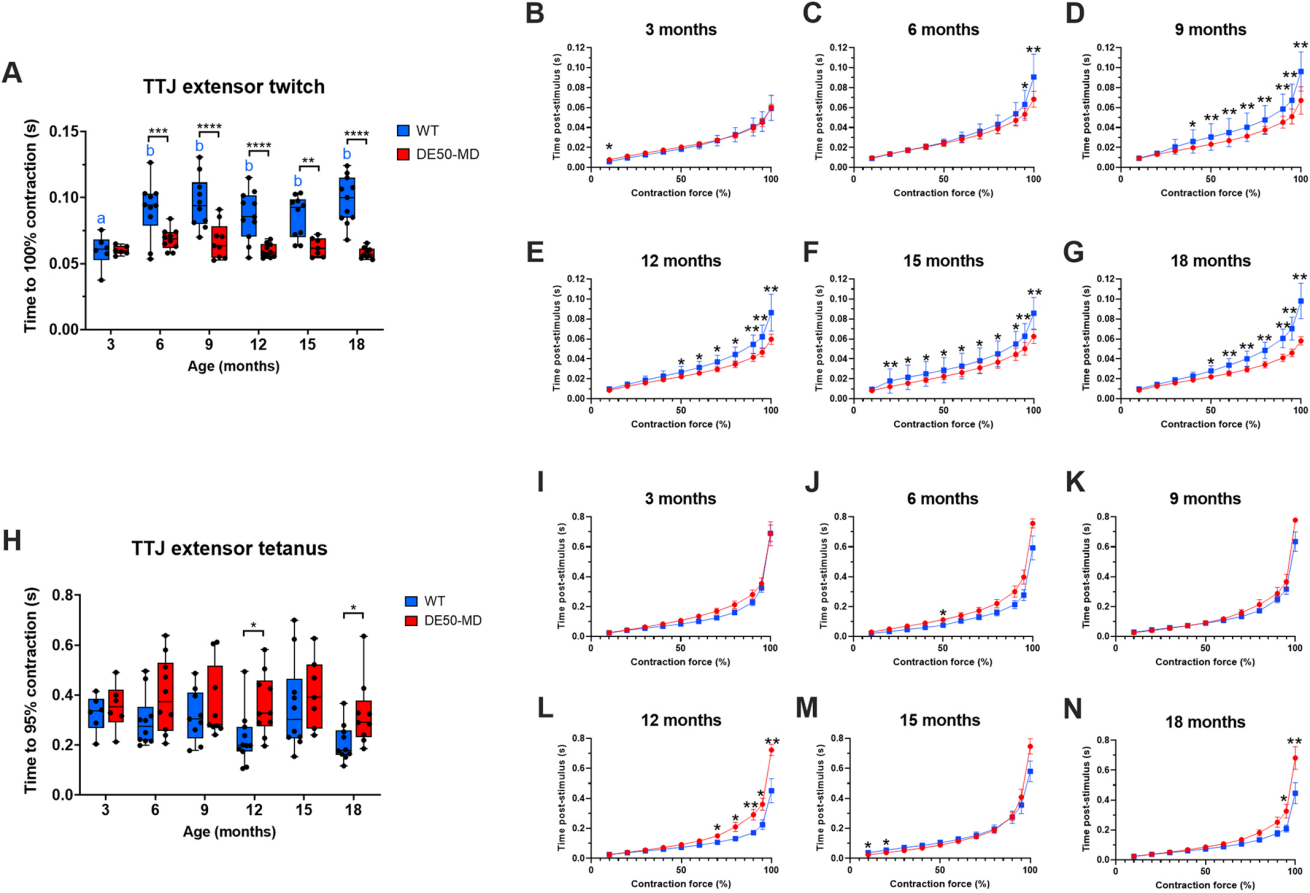
**Contraction time for TTJ extensors.** Comparison of WT (blue) and DE50-MD (red) dogs studied longitudinally from 3 to 18 months of age showing: (A) time to maximum contraction for TTJ extensor twitch; (B-G) time course for contraction for TTJ extensor twitch in 10% increments; (H) time to 95% contraction for TTJ extensor tetanus; and (I-N) time course for contraction for TTJ extensor tetanus in 10% increments. For all graphs, *n*=5-11 WT dogs or 6-9 DE50-MD dogs. Asterisks denote the levels of significance of differences between DE50-MD and WT genotypes based on linear mixed model and post hoc analysis with Holm–Šídák correction for multiple comparisons: *P<0.05; **P<0.01; ***P<0.001; ****P<0.0001. Letters denote differences in the mean within the DE50-MD (red) or WT (blue) genotype; means sharing a letter are not significantly different.

The same pattern was not seen for TTJ extensor data: time to reach maximum force for TTJ extensors was greater in WT dogs compared to that in DE50-MD dogs at all ages except at 3 months (Fig. 5A, *P*<0.01). Analysis of contraction in 10% increments showed that at 3 and 6 months of age, the contraction time course was the same for both genotypes, with the exception of the final 10% of force development in 6-month-old dogs, where WT dogs took longer to reach peak contraction (Fig. 5B,C). From 9 to 18 months, WT dog muscles typically took longer to contract throughout the time course and the difference between genotypes diverged, showing maximal differences in the terminal phase of contraction (Fig. 5D-G). In contrast to TTJ extensor twitch results, time to reach 95% contraction for TTJ extensor tetanus was longer for DE50-MD dogs compared to that for WT dogs (*P*=0.001; Fig. 5H), however post hoc analysis showed that differences within ages were only significant at 12 and 18 months. The contraction time course in 10% increments showed that at 12 and 18 months, both genotypes initially contracted at similar rates before diverging, and DE50-MD dogs took longer to reach 70-100% and 95-100% contraction, respectively (Fig. 5L,N). At all other timepoints (3, 6, 9 and 15 months), TTJ extensor tetanus contraction time courses overlapped for the two genotypes (Fig. 5I-K,M; see Table S4 for TTJ flexor and extensor time to contraction summary data).

#### Relaxation dynamics

Following nerve stimulation, the torque-time traces for several DE50-MD and WT dogs did not return to baseline within the 0.8 s recording period. This more commonly occurred following tetanic contractions. This could be attributed to the higher force produced by this form of contraction and consequent longer relaxation period (exceeding the 0.8 s recording period) or to technical reasons: slight movement of the force-transducing foot pedal, induced by the force of contraction, could account for the trace not returning perfectly to baseline. For this reason, the time taken to reach 95% relaxation was used as a proxy for full relaxation.

**Fig. 6.**
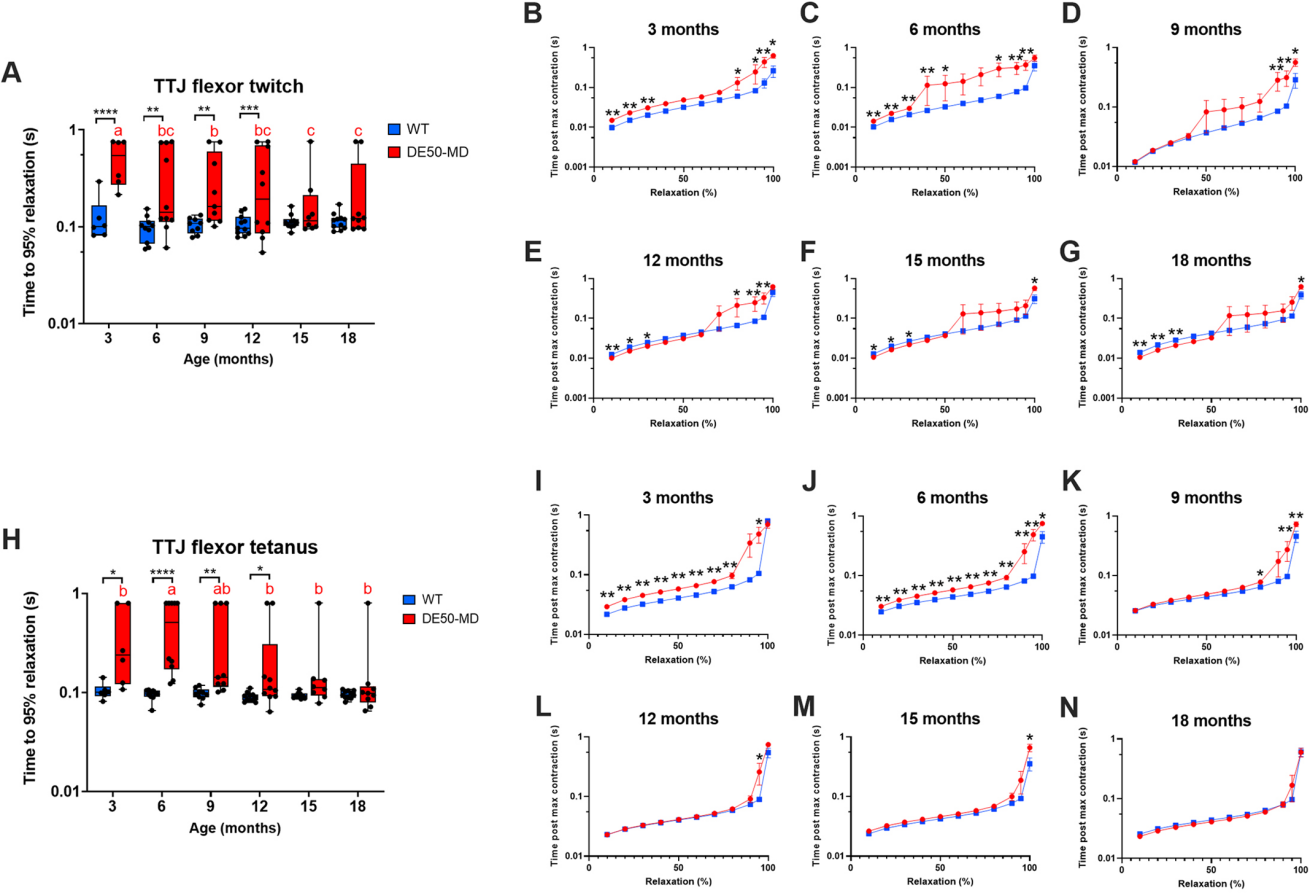
**Relaxation time following TTJ flexor twitch and tetanus.** Comparison of WT (blue) and DE50-MD (red) dogs studied longitudinally from 3 to 18 months of age showing: (A) time to 95% relaxation following TTJ flexor twitch; (B-G) time course for relaxation following TTJ flexor twitch in 10% increments; (H) time to 95% relaxation following TTJ flexor tetanus; and (I-N) time course for relaxation following TTJ flexor tetanus in 10% increments. For all graphs, *n*=5-11 WT dogs or 6-9 DE50-MD dogs. Asterisks denote the levels of significance of differences between DE50-MD and WT genotypes based on linear mixed model and post hoc analysis with Holm–Šídák correction for multiple comparisons: *P<0.05; **P<0.01; ***P<0.001; ****P<0.0001. Letters denote differences in the mean within the DE50-MD (red) or WT (blue) genotype; means sharing a letter are not significantly different.

The time taken to reach 95% relaxation following nerve stimulation was prolonged in DE50-MD dogs compared to that in WT controls for TTJ flexor twitch (*P*=0.001; Fig. 6A) and tetanus (*P*=0.007; Fig. 6H). For both TTJ flexor twitch and tetanus, 3- and 6-month-old DE50-MD dogs typically took longer than age-matched WT dogs to relax throughout the relaxation time course (10-100%) (Fig. 6B,C,I,J); however, some DE50-MD dogs had a very prolonged final 50% relaxation following TTJ flexor twitch (Fig. 6B,C). From 12 to 18 months of age, both groups of dogs initially relaxed at similar rates until the terminal phase of relaxation (95-100%), for which DE50-MD dogs typically took longer to achieve full relaxation (Fig. 6D-G,K-N).

**Fig. 7.**
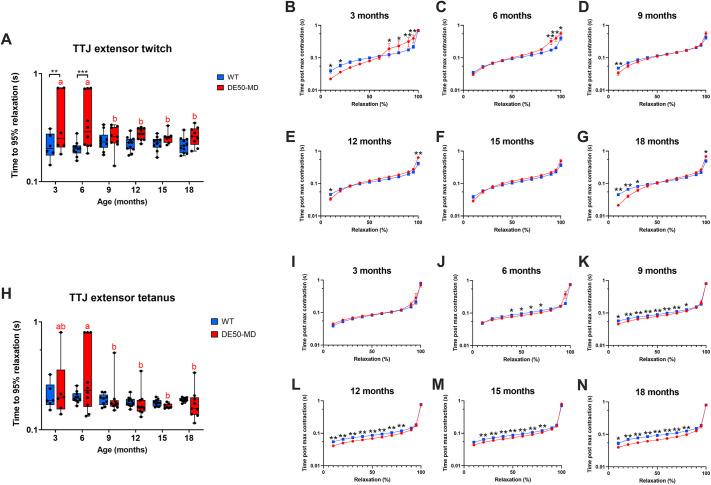
**Relaxation time following TTJ extensor twitch and tetanus.** Comparison of WT (blue) and DE50-MD (red) dogs studied longitudinally from 3 to 18 months of age showing: (A) time to 95% relaxation following TTJ extensor twitch; (B-G) time course for relaxation following TTJ extensor twitch in 10% increments; (H) time to 95% relaxation following TTJ extensor tetanus; and (I-N) time course for relaxation following TTJ extensor tetanus in 10% increments. For all graphs, *n*=5-11 WT dogs or 6-9 DE50-MD dogs. Asterisks denote the levels of significance of differences between DE50-MD and WT genotypes based on linear mixed model and post hoc analysis with Holm–Šídák correction for multiple comparisons: *P<0.05; **P<0.01; ***P<0.001. Letters denote differences in the mean within the DE50-MD (red) or WT (blue) genotype; means sharing a letter are not significantly different.

The time taken to reach 95% relaxation following nerve stimulation was also prolonged in DE50-MD dogs compared to that in WT controls for TTJ extensor twitch (*P*=0.02; Fig. 7A) but not for TTJ extensor tetanus (*P*=0.078) (Fig. 7H). In contrast to relaxation following TTJ flexor contraction, following TTJ extensor twitch, DE50-MD dogs initially relaxed more rapidly than WT dogs following maximum contraction. However, beyond 50% relaxation, the rate of relaxation of DE50-MD dogs was equal to or slower than that of WT dogs (Fig. 7B-G). Following TTJ extensor tetanus, young DE50-MD and WT dogs (3-6 months of age) relaxed at similar rates throughout the time course, whereas 9- to 18-month-old DE50-MD dogs relaxed more rapidly than WT dogs from 10-80% of the relaxation time course. From 90% relaxation onwards, the traces converged and, regardless of genotype, the torque trace did not completely return to baseline within the 0.8 s recording window for most dogs (Fig. 7I-N; see Table S5 for TTJ flexor and extensor time to contraction summary data).

### EC decrement protocol

Following the twitch and tetanus protocol for both the TTJ flexors and extensors, the TTJ flexors were stimulated in an EC decrement (ECD) protocol: an isometric tetanic contraction was induced and following 0.5 s of contraction, the foot pedal was rotated by 29° over a 0.2 s period, causing extension of the TTJ and thereby eccentric muscle stretch of approximately 15% during TTJ flexor muscle contraction (Fig. S3). This was repeated ten times, with a 4 s gap between contractions. The sequence of ten stimulations was repeated an additional two times, with a 4 min rest period between sequences, for a total of 30 ECs. The force produced during the isometric plateau phase of each tetanic contraction was divided by the maximum isometric tetanic force produced during the protocol to calculate the ECD.

**Fig. 8.**
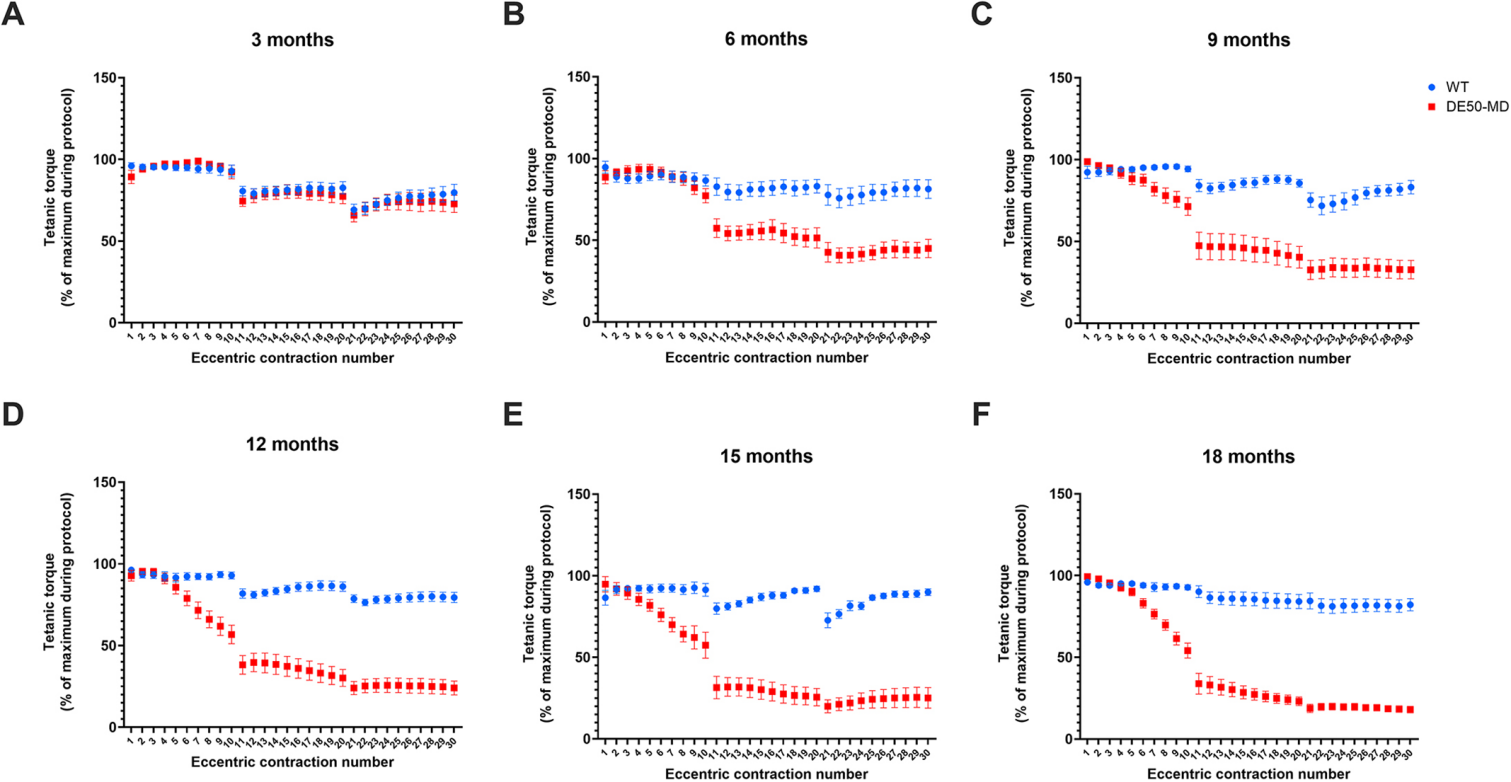
**ECD during a 30-EC protocol.** Comparison of mean WT (blue) and DE50-MD (red) TTJ flexor tetanic torque throughout a series of 30 ECs, displayed as a percentage of the maximum torque produced during the protocol. Contractions were carried out in three groups of ten, with a 4 s gap between individual stimulations, and a 4 min gap between each group of ten stimulations. Dogs were studied longitudinally at (A) 3 months (WT, *n*=6; DE50-MD, *n*=6), (B) 6 months (WT, *n*=8; DE50-MD, *n*=10), (C) 9 months (WT, *n*=8; DE50-MD, *n*=8), (D) 12 months (WT, *n*=11; DE50-MD, *n*=8), (E) 15 months (WT, *n*=9; DE50-MD, *n*=9) and (F) 18 months (WT, *n*=9; DE50-MD, *n*=6). Error bars represent s.e.m.

**Fig. 9.**
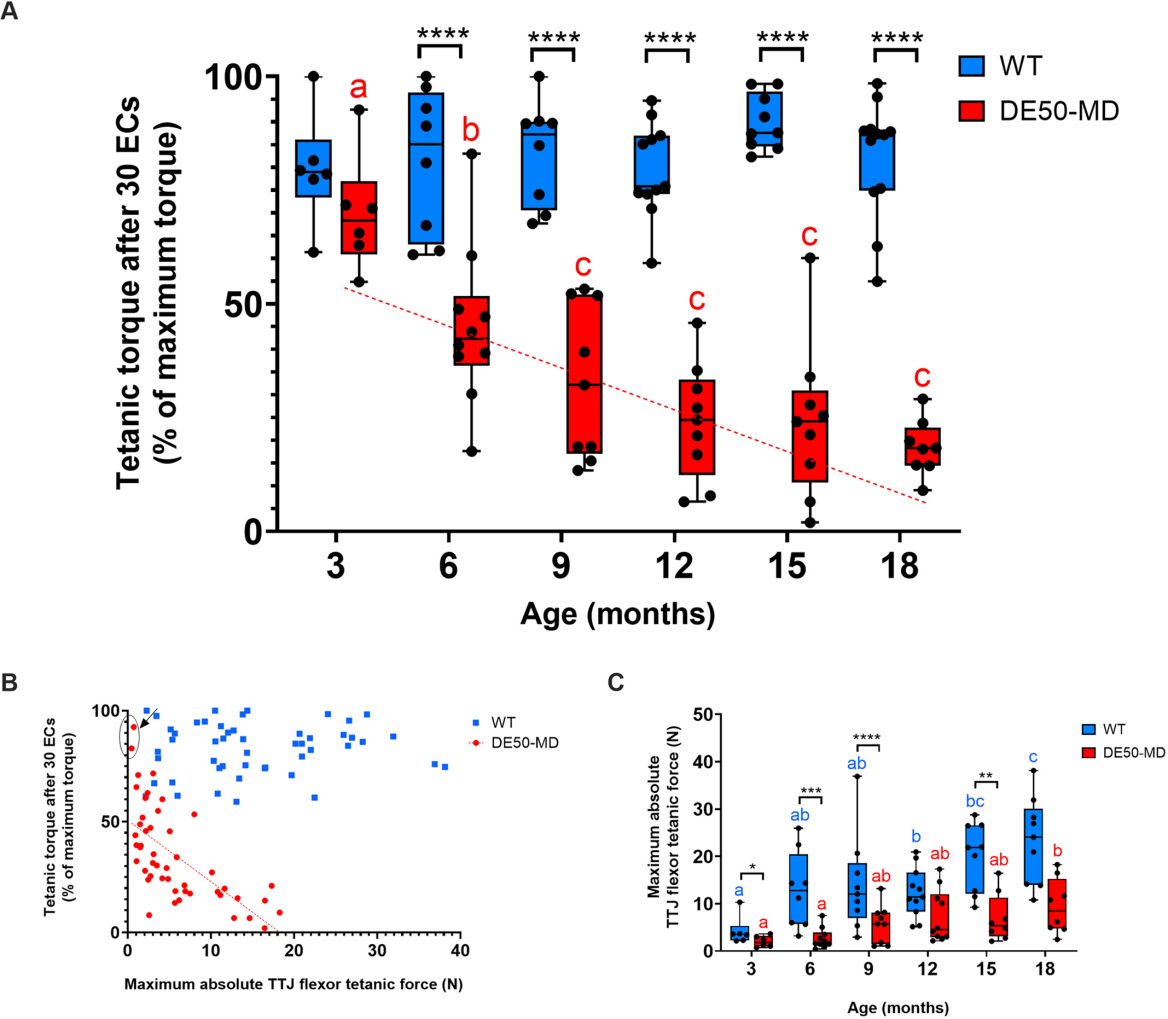
**ECD and correlation with maximum force during a 30-EC protocol.** (A) ECD of the final TTJ flexor tetanus as a percentage drop from the maximum tetanic torque produced during the ECD protocol. The red dotted line shows the relationship between age and torque loss after 30 ECs (percentage drop from the maximum tetanic torque during the protocol). There was a negative correlation between the two variables in DE50-MD dogs (red; linear regression: R^2^=0.53, *P*<0.0001; *n*=51 datapoints from 16 dogs). No correlation was seen in WT dogs (blue; linear regression: *P*=0.43; *n*=53 datapoints from 14 dogs). Within WT dogs, there was no difference in ECD between age groups. (B) Relationship between maximum absolute TTJ flexor force (N) and torque remaining after 30 ECs (percentage of maximum tetanic torque during the protocol). There was a negative correlation between the two variables in DE50-MD dogs (red circles; linear regression: R^2^=0.51, *P*<0.0001; *n*=48 datapoints from 14 dogs). No correlation was seen in WT dogs (blue squares; linear regression: *P*=0.64; *n*=50 datapoints from 12 dogs). The two circled datapoints represent the DE50-MD dogs Y1 and U1, which had minimal torque loss compared to other DE50-MD dogs in their age groups. (C) Maximum TTJ flexor tetanic force (N) during the ECD protocol for WT and DE50-MD dogs. Asterisks denote the levels of significance of differences between DE50-MD and WT genotypes based on linear mixed model and post hoc analysis with Holm–Šídák correction for multiple comparisons: *P<0.05; **P<0.01; ***P<0.001; ****P<0.0001. Letters denote differences within the DE50-MD genotype; means sharing a letter are not significantly different.

Following 30 ECs, WT dogs aged 3 to 18 months had a torque loss of 17±11% (mean±s.d.) and the magnitude of this decrement was unaffected by age (*P*=0.38; Figs 8A-F and 9A). At 3 months of age, there was no significant difference in torque loss between WT and DE50-MD dogs (*P*=0.45). However, from 6 months onwards, the decline in torque was significantly greater for DE50-MD dogs than that for WT dogs at all ages [*P*<0.0001; Fig. 9A; see Table S6 for ECD protocol summary data]. Over the 18 months, there was a significant negative correlation between age and torque remaining after 30 ECs in DE50-MD dogs (R^2^=0.53, *P*<0.0001) but not in WT dogs (*P*=0.43) (Fig. 9A).

Under the assumption that sarcolemmal stress (and associated dystrophic damage) increases with greater muscle force generation, we hypothesised that the magnitude of torque loss in DE50-MD dogs similarly correlates with maximal absolute force: low maximal force during the ECD protocol might thus elicit more modest declines in torque. As shown in Fig. 9B, there was indeed a negative correlation between the maximum absolute force (N) and the torque remaining following 30 ECs (percentage of the maximum torque produced during protocol) in DE50-MD dogs (R^2^=0.51, *P*<0.0001) but not in WT controls (*P*=0.64). The two dogs that produced contractions with the lowest force also had the smallest percentage of torque loss in our DE50-MD population (dog DE50-Y1 aged 3 months and dog DE50-U1 aged 6 months; Fig. 9B, circled datapoints). Both WT and DE50-MD dogs showed a progressive increase in maximum absolute tetanic force (N) during the ECD protocol with increasing age, commensurate with animal growth (Fig. 9C).

### Sample size and power calculations

To determine which of the metrics evaluated in this study would be applicable to pre-clinical assessment of muscle function in treatment trials, we performed sample size and power calculations (Table 1) for all physiology parameters that significantly differentiated the two genotypes. We quantified the number of study animals required to detect a 100, 75, 50 or 25% improvement in DE50-MD results compared to WT levels. Of all the parameters tested, ECD showed the most power as a biomarker and could detect (with a sample size of two animals per genotype) as little as a 25% improvement in torque loss. Accordingly, this metric had the highest predictive power when using *n*=6 animals. TTJ flexor muscle absolute and relative force, and the time taken to relax following TTJ flexor twitch and tetanic contractions, also showed potential value as biomarkers; however, the sensitivity of these parameters in detecting small improvements in DE50-MD subjects was relatively low. The same was true for TTJ extensor twitch absolute force and time to maximal contraction and relaxation, and for TTJ extensor twitch:tetanus torque ratio.

**Table 1.**
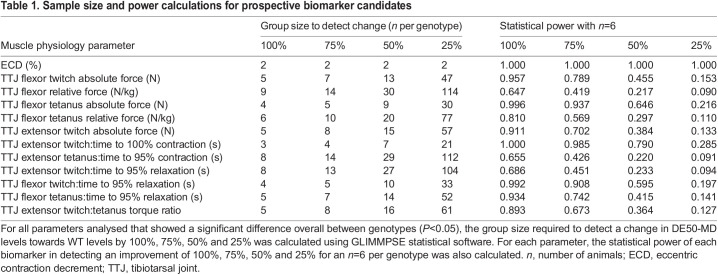
Sample size and power calculations for prospective biomarker candidates

### Fibre typing

**Fig. 10.**
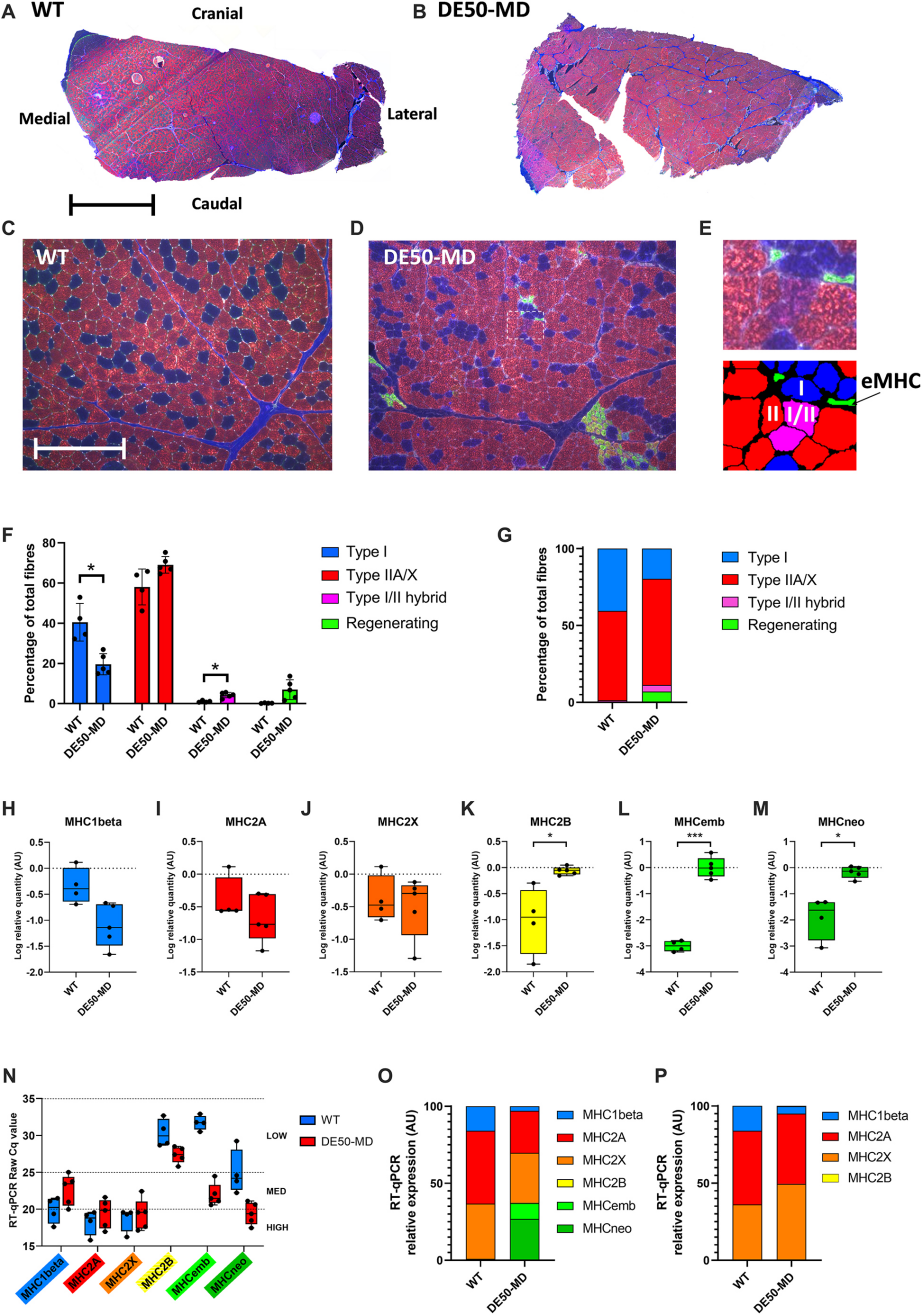
**Fibre typing of the cranial tibial muscle in 18-month-old dogs.** (A,B) Representative images of the entire transverse section of a WT (A) and DE50-MD (B) cranial tibial muscle (primary TTJ flexor). Type I fibres are labelled blue, type IIA and IIX fibres are labelled red, regenerating fibres (MHCemb) are labelled green and collagen VI outlines the fibres in white. Type I/II hybrid fibres appear purple. Orientation of the muscle *in situ* in the pelvic limb of the dog is labelled. Scale bar: 5 mm. (C,D) Representative 10× magnification images of WT (C) and DE50-MD (D) muscle used for ImageJ fibre type quantification. Scale bar: 200 µm. (E) Representative cranial tibial muscle area (magnified view of the dotted box in D) and ImageJ overlay below showing the various muscle fibres categories recognised by ImageJ [type I, type IIA/X, type I/II hybrid and regenerating (MHCemb or eMHC)]. (F) Comparison of cranial tibial fibre type composition (percentage of total fibre number in the visual field) between WT and DE50-MD genotypes (mean result of five images per dog; WT, *n*=4; DE50-MD, *n*=5) analysed by multiple two-tailed unpaired *t*-tests and Holm–Šídák multiple comparison correction. Error bars represent s.d. (G) Fibre type composition (percentage of total number of fibres in the visual field) of cranial tibial muscle sections in WT and DE50-MD genotypes. (H-M) RT-qPCR results showing log relative quantity (normalised to the reference genes *HPRT1*, *RPL13A* and *SDHA*) of gene expression levels for MHC1β (H), MHC2A (I), MHC2X (J), MHC2B (K), MHCemb (L) and MHCneo (M). (N) Comparison of RT-qPCR raw Cq values for MHC1β, MHC2A, MHC2X, MHC2b, MHCemb and MHCneo. Boxes extend from the 25th to 75th percentile, with the lines within the boxes at the median value and whiskers show the minimum and maximum results. (O) Estimated relative gene expression levels of MHC isoforms within each genotype. (P) Estimated relative gene expression levels of MHC isoforms within each genotype excluding regenerative isoforms (MHCemb and MHCneo). Asterisks denote the levels of significance of differences between the DE50-MD and WT genotypes: *P<0.05; ***P<0.001. AU, arbitrary units.

To help explain the differences in contraction and relaxation dynamics between genotypes, multiplex immunofluorescence fibre typing was performed for the primary TTJ flexor, the cranial tibial muscle (Fig. 10A-G; see Table S7 for data summary). The TTJ flexors were selected for further analysis based on ECD, twitch and tetanus sample size and power calculations that suggested that TTJ flexor muscle physiology biomarkers were superior at distinguishing between DE50-MD and WT genotypes compared to the TTJ extensors. Furthermore, the cranial tibial muscle is a well-defined muscle that readily permits staining of the entire transverse section, unlike the TTJ extensor, the gastrocnemius. A surprising finding was that type I fibres were significantly reduced in DE50-MD muscle (*P*=0.01) at 20% of total fibres, compared to 41% in WT muscle (Fig. 10F,G). The percentage of type II fibres (IIA/IIX combined) was concomitantly higher in DE50-MD (mean, 69%; range, 65-75%) compared to that in WT (mean, 58%; range, 46-66%); however, here, the difference did not achieve significance (*P*=0.06). Type I/II hybrid fibres were present in significantly (*P*=0.01) higher numbers in DE50-MD muscle (mean, 4%; range, 2-6%) than in WT muscle (mean, 1%; range, 0.7-1.7%). Regenerating fibres were present in modest numbers in DE50-MD muscle (mean, 7%; range, 1-14%) as expected for 18-month-old dystrophic muscle (Hildyard et al., 2022), but were essentially absent in WT muscle (mean, 0.2%; range, 0.1-0.4%).

To corroborate these findings, we also conducted reverse-transcription quantitative PCR (RT-qPCR) using cDNA prepared from RNA extracted from cranial tibial muscle tissue homogenates. In contrast to fibre typing, these investigations revealed no statistically significant differences between genotypes in relative gene expression of *MYH7* (encoding MHC1β) (*P*=0.06; Fig. 10H), *MYH2* (MHC2A) (*P*=0.47; Fig. 10I) or *MYH1* (MHC2X) (*P*=0.69; Fig. 10J), although MHC1β gene expression was lower for 4/5 DE50-MD dogs than that in WT dogs (see Table S7 for RT-qPCR data summary). Increases were, however, observed in expression of *MYH4* (MHC2B; Fig. 10K). Expression of the regenerative myosin heavy chain (MHC) genes *MYH3* and *MYH8* (encoding MHCemb and MHCneo, respectively) was also markedly elevated in DE50-MD muscle (as reported previously; Hildyard et al., 2022) (Fig. 10L-M).

These comparative metrics, however, do not account for relative abundance between isoforms; we thus compared raw cycle quantification (Cq) values across the different isoforms (Fig. 10N). The most abundantly expressed MHC genes were those encoding MHC2A and MHC2X (raw Cq values of 16-22), with MHC1β gene expression present at lower but still substantial levels (Cq, 18-25). Regenerative MHC genes within dystrophic muscle were also highly expressed (MHCemb Cq, 21-24; MHCneo Cq, 17-21). Overall levels of MHC2B gene expression (Cq, 26-33) were orders of magnitude lower than those of other isoforms, and indeed, canonically, the muscle of large mammals does not contain fast glycolytic MHC2B fibres: the low levels of mRNA measured here suggest that despite the statistically significant increase in expression within dystrophic muscle, MHC2B likely does not meaningfully contribute to the muscle fibre milieu of either genotype. Based on these Cq values, the relative abundance of each transcript was estimated within each genotype (Fig. 10O): collectively, the regenerative MHC genes accounted for ∼40% of total MHC gene expression within dystrophic muscle. We reasoned that the high expression of these regenerative markers would concomitantly lower proportional expression of other MHCs, potentially masking more subtle changes in expression. Accordingly, comparison of the relative expression of non-regenerative myosins only (Fig. 10P) was in agreement with our immunohistochemical fibre-typing data: MHC1β gene expression was proportionally reduced in DE50-MD muscle, and whereas expression levels of the MHC2A gene appeared largely unchanged, MHC2X gene expression levels appeared to be elevated.

## DISCUSSION

Muscle function indicators are crucial outcome measures for muscular dystrophy treatment trials; hence, establishing muscle function biomarkers in the DE50-MD canine model of DMD is fundamental to its preclinical use. Muscle physiology protocols performed under anaesthesia offer a way to quantify muscle function in the absence of confounding factors such as motivation of the test subject. Here, we present the most extensive longitudinal assessment of muscle functional decline in any canine model of DMD, over an age range that is likely applicable to future trials. Notably, the age-associated decline in resistance to eccentric exercise is an excellent objective biomarker in this model.

Indeed, of all the parameters analysed in this study, ECD best discriminated, with very high statistical power, DE50-MD from WT individuals. ECD analysis was performed on the TTJ flexor muscles, based on a protocol established by pioneering work in the GRMD model of DMD (Tegeler et al., 2010). Evaluation of this muscle group allowed measurement of force generation and eccentric stretch to be assessed *in vivo,* under general anaesthesia, multiple times as the animals aged. ECD was significantly greater in DE50-MD individuals than in WT individuals from 6 months of age through to the end of our study at 18 months of age. Percentage losses (after 30 ECs) increased in magnitude between 6 and 9 months of age, but then largely stabilised, with a mean loss of 76±14% (mean±s.d.) of maximum torque being observed from 9 to 18 months of age. Sample size calculations indicate that with only two dogs per genotype, it would be possible to detect as little as a 25% improvement in resistance to ECs with a statistical power of 0.8. Conducting pre-clinical trials in large animal models has many practical and financial challenges. Findings from this paper and others (Hornby et al., 2021; Riddell et al., 2021, 2022; Hildyard et al., 2022) characterising the DE50-MD model have indicated that an *n* of six dogs per genotype would be optimal for a select panel of biomarkers with high statistical power to detect a phenotypic improvement in response to treatment. ECD would thus be an excellent addition to this panel of biomarkers for future pre-clinical trials in the DE50-MD model.

The ECD protocol revealed no difference between genotypes at 3 months of age. In contrast, the GRMD dog model does show an ECD at 3 months of age; however, the magnitude of this decline is modest and is lower than that of 6-month-old GRMD dogs (Kornegay et al., 2014). As DMD is progressive, the lack of ECD in 3-month-old DE50-MD muscle might reflect the milder pathology present at this age. Supporting this, previous work (studying the same cohort of dogs analysed here, at the same 3-monthly intervals) showed lower muscle volumes on MRI in DE50-MD dogs compared to those in WT dogs at all ages with the exception of 3 months (Hornby et al., 2021). Furthermore, the force produced by muscles of younger dogs is markedly lower than that of older animals, potentially lessening the severity of eccentric stress on the contracting muscle fibres. Indeed, in DE50-MD dogs, we observed a positive correlation between maximum absolute force produced and the magnitude of ECD, and this finding has been reported in previous studies (McCully and Faulkner, 1986; Warren et al., 1993). An association between growth and increasing load per muscle fibre has previously been modelled and correlated with the observed greater severity of DMD in larger animal models, such as the GRMD dog model, compared to smaller animal models, such as the *mdx* mouse model (Bodor and McDonald, 2013). The smaller size of DE50-MD dogs compared to that of GRMD dogs of the same age might thus explain why an ECD is seen in GRMD dogs but not DE50-MD dogs at 3 months of age.

TTJ flexor and extensor isometric force analysis also revealed some significant differences between WT and DE50-MD dogs. Although statistical significance values and sample size calculations suggest that these measures were of lower utility as biomarkers than ECD, the results provide a better understanding of the DE50-MD phenotype, from which we can derive mechanistic insight. Older DE50-MD dogs typically had lower absolute and bodyweight-corrected (relative) force compared to those of age-matched WT dogs, with greater differences between genotypes for the TTJ flexors (innervated by the fibular nerve) than for the extensors (innervated by the tibial nerve). Both genotypes exhibited comparable isometric force at 3 months of age, unlike the GRMD dog model, which has significantly reduced TTJ flexor and extensor isometric force at 3 months (Kornegay et al., 1999). The similarity between genotypes at 3 months aligns with our ECD protocol findings and could be explained by the same reasonings discussed in the previous paragraph, namely, milder pathology and/or small body size. The twitch:tetanus ratio was significantly reduced in DE50-MD dogs for TTJ extensor contraction but not for TTJ flexor contractions, indicating that twitch torque was impaired to a greater extent than tetanic torque in the TTJ extensor muscles, whereas both twitch and tetanic torque were proportionately affected in the TTJ flexors.

Reduced absolute and relative isometric TTJ muscle force in DE50-MD dogs suggests that age-matched dystrophic dogs typically produce both less force overall and less force per unit of muscle. Absolute isometric muscle force is affected by both muscle mass and the force-generating capacity of the muscle, whereas specific force adjustment defines muscle strength independent of muscle size (Yang et al., 2012). Lower absolute TTJ flexion and extension force in DE50-MD dogs could, in part, be attributed to their reduced muscle mass compared to that of age-matched controls; although DE50-MD dogs are typically a similar height as that of WT dogs, they have lower bodyweights and muscle volumes compared to those of WT dogs as shown by MRI (Hornby et al., 2021). In terminal muscle physiology studies, post-mortem dissection allows specific force to be calculated by normalising absolute force to the cross-sectional area of the stimulated muscles; however, due to the longitudinal nature of our study, this was not possible. Consequently, to account for differences in the body mass of dogs, absolute force was corrected to bodyweight to give the relative force. This method of normalisation is imperfect as bodyweight is influenced by multiple factors, including muscle weight and overall dog size. Furthermore, muscle weight itself does not represent only muscle tissue mass: muscles in dystrophic animals have higher fluid and collagen content compared to those in healthy animals (Garlich et al., 2010), further complicating normalisation and confounding comparisons of isometric torque between genotypes (Call et al., 2011). Nonetheless, normalising to bodyweight is straightforward and, accordingly, is the accepted method for assessment of muscle force in dogs (https://www.treat-nmd.org/wp-content/uploads/2023/07/GRMD-DMD_D.2.2.001.pdf). Normalisation to bodyweight reduced isometric force differences between genotypes, but statistical significance was retained for most age groups, suggesting that DE50-MD muscles are indeed weaker than size-matched WT muscles.

Analysis of torque dynamics showed that DE50-MD dogs typically had faster contraction and prolonged relaxation times compared to those of WT dogs. Contraction time was generally longer for WT dogs compared to that for DE50-MD dogs for isometric twitch; however, DE50-MD TTJ flexor twitches took significantly longer to reach peak contraction at 3 and 6 months of age. Increased contraction times were also reported in the GRMD dogs at 3 and 6 months of age for the TTJ flexor muscles (Kornegay et al., 1994), whereas reduced contraction times have been reported for the extensor carpi ulnaris (ECU) of a 3-year old DMD dog model (Hakim et al., 2021). Studying the torque development in 10% increments showed differences between genotypes, muscle groups and stimulation type; however, typically the torque-time trace overlapped between genotypes in the early stages and diverged towards the end of contraction. The time between electrical stimulation and onset of torque production is referred to as the electromechanical delay, as it can be affected by differences in electrochemical processes (primarily synaptic transmission and excitation-contraction coupling) and by mechanical components of force transmission (Lacourpaille et al., 2014). Studies have suggested that although the quantity of calcium ion release from the sarcoplasmic reticulum is reduced in DMD, the time taken to release the maximal quantity is not different between dystrophic and healthy muscle (Woods et al., 2005; Lacourpaille et al., 2014). Rather, differences in time to contract are primarily linked to structural differences (Lacourpaille et al., 2014). Rapid contraction is characteristic of fast muscle fibre types (Moran et al., 2005). It has been widely reported that in dystrophic muscle, fast fibres are preferentially affected and are eventually lost, leading to a fast-to-slow transition (Webster et al., 1988; Pedemonte et al., 1999). However, as noted above, a recent study showed a reduced time to peak contraction in conjunction with a slow to fast fibre shift in the ECU muscle of a canine model of DMD compared to that in WT dogs (Hakim et al., 2021). Furthermore, a study using the CXMD_J_ canine model of DMD showed that fibre type composition of the cranial tibial and diaphragm in affected dogs was both age and muscle dependent (Yuasa et al., 2008).

To establish whether fibre type alterations might be contributing to our muscle physiology results, fibre typing was performed for the primary TTJ flexor, the cranial tibial muscle. This anatomically well-defined muscle allowed staining and analysis of the entire transverse section (although owing to the longitudinal nature of the study, obtaining this muscle was only possible post-mortem at the 18-month timepoint). Contrary to our expectations, we found that the cranial tibial muscle of DE50-MD dogs exhibited a modest but consistent slow-to-fast shift (and concomitant enrichment of slow-fast hybrid fibres) compared to that of WT dogs. This finding is opposed to widely reported fast-to-slow fibre shifts and the general understanding that type II fast fibres are preferentially lost in dystrophic individuals (Webster et al., 1988), but corroborates recent results published on the ECU muscle of another dog model of DMD (Hakim et al., 2021) as well as our finding of faster twitch contraction velocity in 18-month old DE50-MD dogs. In contrast, a paper studying the CXMD_J_ canine model of DMD found an increased proportion of slow fibres in the cranial tibial muscle of a single affected animal compared to that in a single WT dog at 12 months of age (Yuasa et al., 2008). The fact that DE50-MD muscle had longer contraction times at early timepoints, whereas older muscle showed shorter contraction times, indicates that the slow-to-fast shift in fibre type is age specific, occurring in relatively older dystrophic animals. Investigation of this hypothesis, however, would require biopsy of the DE50-MD cranial tibial muscle at multiple timepoints, which was not possible within the remit of this longitudinal study. These findings suggest that, at least in some dog models of DMD, an unexpected slow-to-fast fibre type transition does indeed occur in certain muscles. Our findings challenge the conventionally held, fast-to-slow fibre shift model and imply that fibre type shifts in dystrophic muscle should not be generalised or assumed in mammals.

In addition to a slow-to-fast fibre type shift, we also observed increased numbers of regenerating fibres and increases in MHCemb, MHCneo and MHC2B gene expression in DE50-MD muscle. The modest elevation in regenerating fibres and expression of MHCemb and MHCneo observed in the 18-month-old DE50-MD cranial tibial muscles is in agreement with previous work demonstrating acute muscle regeneration in DE50-MD dogs between 3 and 18 months of age, with the peak degenerative/regenerative period occurring earlier, at 6-9 months (Hildyard et al., 2022). The increase in MHC2B gene expression in DE50-MD muscle is intriguing: similar to humans, this MHC is not considered to be canonically expressed within canine skeletal muscle (Toniolo et al., 2007). We note, however, that statistical significance does not equate to biological significance, and, in both healthy and DE50-MD muscle, gene expression levels of MHC2B remained orders of magnitude lower than those of all other MHC isoforms (including MHCemb and MHCneo in dystrophic muscle). Indeed, when relative gene expression levels of the MHCs were compared, our RT-qPCR data suggested that MHC2B transcripts accounted for only 0.1% of total MHC transcripts in DE50-MD dogs and just 0.02% in WT dogs. Consequently, MHC2B is highly unlikely to contribute to contractile behaviour in the muscle of either genotype, and this finding likely represents dysregulation of the otherwise tightly controlled myosin expression program, rather than a physiological response to dystrophin deficiency.

A longer relaxation phase was often observed following maximal contraction in DE50-MD dogs compared to that in WT dogs. This feature was more common following stimulation of the TTJ flexors than the extensors. Similar to the contraction dynamics, the early stages of muscle relaxation progressed at different rates between muscle groups, genotypes and within genotypes of different age groups; however, typically the terminal stages of relaxation were prolonged in DE50-MD dogs across the age groups. A similar biphasic pattern has previously been seen in GRMD dogs (Kornegay et al., 1994), with initial relaxation occurring at the same speed as WT dogs and then slowing greatly. In dystrophic muscle, during the relaxation phase of contraction, there is accelerated clearance of calcium, but complete clearance is not achieved, which can cause persistent muscle tone (Hughes et al., 2019). Although differences in muscle fibre type composition can also contribute to prolonged relaxation times, our fibre typing results showed a slow-to-fast fibre type shift in the DE50-MD TTJ flexors, whereas longer relaxation is characteristic of type I (slow twitch) fibres (Wiles et al., 1979). Alternative dystrophic changes seen histologically in DE50-MD muscle that could contribute to a prolonged relaxation time include increased endomysial fibrosis and inflammatory infiltration (Hildyard et al., 2022). As the extracellular matrix is critical for the transmission of muscle force to tendons and contributes to muscle force production via passive viscoelastic mechanisms (Silver and Landis, 2008), changes in its composition in dystrophic muscle might impact the relaxation phase of muscle contraction.

In summary, this work has identified TTJ flexor muscle ECD as an excellent biomarker of the DE50-MD phenotype between the ages of 6 and 18 months, with great potential for identifying a response to treatments in future pre-clinical trials. In addition, we have shown that DE50-MD TTJ flexor muscles have reductions in force-generating capacity, faster contraction time in older dogs in conjunction with a slow-to-fast fibre type shift, and prolonged muscle relaxation following contraction, compared to those of age-matched WT dogs. DE50-MD TTJ extensor muscles also showed these muscle physiology alterations, although to a lesser extent. Interestingly, unlike the GRMD model (Kornegay et al., 1994; Tegeler et al., 2010; Childers et al., 2012), for which muscle contractile differences can be detected in dogs as young as 3 months of age, DE50-MD dogs instead showed no difference in absolute or relative force production, and no significant impairment in resistance to ECs at this age.

Our findings add to the growing body of evidence that the DE50-MD dog model of DMD closely mimics the human disease and is a very promising model for future clinical trials. A fundamental advantage of the DE50-MD dog model over other commonly used animal models is that it has a mutation in the most-commonly mutated region of the dystrophin gene in human patients, making it a particularly suitable model for gene therapies targeting this region. In addition, the DE50-MD dog is relatively small compared to other large animal models of the disease, making it more cost effective to house, maintain and treat during pre-clinical trials. The work presented in this paper forms part of an extensive natural history study for work on this dog model, which followed a large number of dystrophic animals alongside their WT littermates for 18 months. This huge undertaking produced a wealth of information regarding the progression of disease in this model, which includes already published findings on blood biomarkers (Riddell et al., 2021, 2022), skeletal muscle histological phenotype (Hildyard et al., 2022) and skeletal muscle MRI (Hornby et al., 2021), all of which support the similarity of the model to the human phenotype. The results of this study confirm that muscle function is impaired in DE50-MD dogs. Muscles of DE50-MD dogs also show reduced absolute force compared to those of age-matched WT dogs, in accordance with MRI findings from previous work that showed DE50-MD dogs had reduced muscle volumes (Hornby et al., 2021), as opposed to the compensatory hypertrophy seen in *mdx* mice (Lynch et al., 2001).

To conclude, this work is a comprehensive evaluation of EC-induced torque decline and isometric contraction torque, force and dynamics in a large number of DE50-MD dogs, examined longitudinally over an 18-month age span. In particular, it highlights the value of ECD as a powerful, non-invasive, objective marker of skeletal muscle function in DE50-MD dogs, with particular promise for its use in long-term treatment trials. Overall, this study comprises the most comprehensive longitudinal study of muscle function in any large animal model of DMD to date, allowing us to make highly informed decisions regarding the design of future pre-clinical trials.

## MATERIALS AND METHODS

### Animal husbandry

Dogs (*Canis familiaris*) used in this study were from the DE50-MD colony, housed in a dedicated facility at the Royal Veterinary College, London, as described previously (Riddell et al., 2021). WT dogs were housed with DE50-MD dogs (according to temperament and hierarchy) in indoor kennels (12 h/12 h light/dark cycle, 15-24°C). The kennel size was 4.5-7.5 m^2^. The Animal (Scientific Procedures) Act 1986 (ASPA) code of practice (Section 2) regarding the number of dogs per area of kennel size was followed; typically, study dogs were housed in groups of two to three. They had daily access (generally from 08:00 to 15:00) to large outdoor paddocks (approximately 100 m^2^) in group sizes of up to five dogs – conditions that exceed the minimum stipulations of ASPA and according to approval by the local Animal Welfare Ethical Review Body at the Royal Veterinary College. To produce all dogs for this study, carrier female Beagle (RCC strain, Marshall Bioresources)-cross dogs, derived from an original founder Bichon-Frise cross Cavalier King Charles Spaniel female carrier, were mated with male Beagles (RCC strain). Pregnant females (single housed) whelped naturally and puppies were kept with the mother in a large kennel with a heat lamp (∼28°C) to allow nursing. All puppies were microchipped at 7 days of age, with cheek swabs collected concurrently for genotyping via PCR/Sanger sequencing (as described previously; Hildyard et al., 2022). Puppies were reared by their mother until approximately 4 weeks, after which they were transitioned to puppy food (Burns) suitable to requirements: puppies under 6 months of age were fed at least three times daily initially with Royal Canin Puppy ProTech Colostrum+Milk mixed with Burns Puppy Original Chicken and Rice, and transitioning to Burns Puppy Original Chicken and Rice mixed with Royal Canin Gastro-Intestinal Puppy Food (2:1). From 6 months onwards, dogs received two feeds daily of Burns Puppy Original Chicken and Rice mixed with Royal Canin Gastro-Intestinal Puppy Food (2:1) and *ad lib* water. Dogs received daily human interaction and underwent a comprehensive socialisation programme. All dogs not required for studies were rehomed.

### ARRIVE guidelines

Animal Research: Reporting of *In Vivo* Experiments (ARRIVE) guidelines were followed for the design and conduct of the study. All experimental procedures involving animals in this study were conducted according to UK legislation, within a project licence (P9A1D1D6E, granted 11 June 2019) assigned under ASPA and approved by the Royal Veterinary College Animal Welfare Ethical Review Body. All efforts were made to minimise any animal suffering throughout the study. Pre-determined endpoints for DE50-MD dogs were established including dehydration (unresolved by fluid treatment), lethargy/motor dysfunction, weight loss/dysphagia, dyspnoea, listless behaviour/demeanour or heart failure. Dogs were observed daily by animal technician staff and animals showing any of these signs were reported to and assessed by the Study Director, the Named Veterinary Surgeon and the Named Animal Care and Welfare Officer. Any dogs reaching a pre-determined endpoint prior to the planned 18-month study end were humanely euthanised. Euthanasia was performed using an overdose of sodium pentobarbital (250 mg/kg, Dolethal, Covetrus) administered intravenously via preplaced catheter.

### Study population

In total, data from 16 DE50-MD and 14 WT dogs are included in this study (Fig. 1). The sample size for the study was determined in order to generate an accurate estimation of the variance for each timepoint and genotype to enable sample size calculations for future studies, as reported in this paper. Dogs were studied longitudinally at 3-monthly intervals, from 3 to 18 months of age. Data were not available for all dogs for all timepoints due to either apparatus unavailability, scheduling conflicts, technical issues with data collection/quality control, or early animal euthanasia (six animals; see Fig. 1). Of the 16 DE50-MD dogs included in this study, ten were euthanised at the end of the planned 18-month study period, five were euthanised prior to the study end as a result of reaching pre-determined humane endpoints (all related to dysphagia – no other humane endpoint was reached during this study), and one (DE50-G4) was euthanised at 11 months of age due to developmental elbow dysplasia, believed to be unrelated to the DMD phenotype. Four of the 14 WT dogs included in this study were euthanised humanely at the end of the planned 18-month natural history study period (WT-G2, WT-J1, WT-K4 and WT-M2), and one additional WT dog (WT-K5) was euthanised at 14 months of age due to developing steroid-responsive meningitis, a condition known to affect the Beagle breed (Lau et al., 2019). All remaining nine WT dogs were rehomed.

### General anaesthesia

Dogs underwent general anaesthesia consisting of pre-medication with intravenous methadone (0.2 mg/kg, Comfortan, Dechra) prior to induction of anaesthesia with intravenous propofol (1-4 mg/kg, PropoFlo Plus, Zoetis), and maintenance with inhaled sevoflurane (1.5-3.5%, SevoFlo, Zoetis) in oxygen administered via a Datex-Ohmeda Aestiva/5 anaesthesia machine. To reduce the need for separate general anaesthetic regimens, this work was conducted alongside vastus lateralis muscle biopsy collection (results of which are reported elsewhere; Hildyard et al., 2022): biopsy samples were collected from the left pelvic limb prior to the muscle physiology protocol (conducted on the right pelvic limb). All dogs received perioperative intravenous cefuroxime antibiotic (20 mg/kg, Zinacef, GSK). For pain relief, a single intravenous dose of carprofen (2 mg/kg, Rimadyl, Pfizer) was given perioperatively, followed by postoperative oral carprofen once daily for 3 days (2 mg/kg, Rimadyl, Pfizer). Body temperature was maintained at approximately 37°C, by the use of an electric heat-pad and a warming blanket (Bair Hugger, Patient Warmer 505).

### Muscle torque apparatus

The muscle physiology system consisted of a large animal frame (Aurora Scientific) with a force-transducing foot pedal and dual-mode lever system (310C-FP, Aurora Scientific) (Fig. S1), connected to a computer with Dynamic Muscle Control (DMC) software (Aurora Scientific, 610A, v5.500). The muscle torque procedure was conducted with the dog under general anaesthesia, lying in dorsal recumbency, positioned with the aid of blankets and sandbags. The tibiotarsal (ankle), stifle (knee) and coxofemoral (hip) joints were positioned at 90° angles. The tarsus was taped with bandaging wrap (Vetrap, 1410) to the force-transducing foot pedal of the musculoskeletal testing rig (Fig. S1A). The foot pedal was attached to a tri-axis mounting frame allowing adjustments to ensure the correct alignment of the joints and positioning of tarsus on the foot pedal for each individual animal (Fig. S1B-E). The cranial aspect of the thigh rested against a height-adjustable foam pad (Fig. S1E). TTJ extension and flexion were induced by supramaximal stimulation of either the tibial or fibular nerves, respectively, via percutaneous placement of two 25 mm length, 27-gauge monopolar needle electrodes (Chalgren, 111-725-24TP), controlled by the DMC software. The resultant force exerted on the foot pedal was recorded by the DMC software.

### Twitch and tetanus protocols

Twitch and tetanus isometric torque protocol and nerve stimulation parameters were selected based on the TREAT-NMD SOP DMD_D.2.2.001 (https://www.treat-nmd.org/wp-content/uploads/2023/07/GRMD-DMD_D.2.2.001.pdf) and work by Tegeler et al. (2010) that established an *in vivo* muscle physiology protocol for dogs. The tibial and fibular nerves were individually supramaximally stimulated via percutaneous electrodes, firstly with a single pulse (twitch) of 150 V lasting 0.1 ms, and secondly by a tetanic run of fifty 150 V pulses over a 1 s period (50 Hz). Needles were not moved between each twitch and tetanus. Two sets of twitch and tetanus were recorded and the set with the highest torque was used for further analysis. Contraction torque and dynamics were quantified by Dynamic Muscle Analysis (DMA) software (Aurora Scientific, 611A, v5.300). The software provided a measure of torque in Newton metres (N m). Absolute force (N) was calculated by dividing torque by the length of the lever arm (m), measured as the length of the tarsus that was in contact with the foot pedal, between the axis of TTJ rotation to the end of the middle digit, and was measured manually with a ruler. Specific force is typically calculated by normalising absolute force to the cross-sectional area of the stimulated muscles; however, owing to the longitudinal nature of our study, this was not possible. Consequently, to account for differences in the body mass of dogs, absolute force (N) was corrected to bodyweight (kg), henceforth termed the relative force (N/kg), as recommended (https://www.treat-nmd.org/wp-content/uploads/2023/07/GRMD-DMD_D.2.2.001.pdf). Traces of muscle torque output from a twitch stimulus typically showed a steep rise to peak contraction, followed by a return of the trace to baseline as the muscle relaxed (Fig. S2A). Induction of a tetanus produced a steep rise in the torque trace, followed by a plateau phase of sustained muscle contraction until the tetanic run of pulses ended, with subsequent return of the torque trace to baseline (Fig. S2B). Certain exclusion criteria were applied to twitch and tetani data upon quality control analysis of the force-time traces; in several cases, abnormal trace conformation precluded accurate force-time analyses. This likely occurred due to aberrant stimulation of muscle groups other than the intended target (see ‘Twitch and Tetani All Data’ at https://doi.org/10.6084/m9.figshare.23501319 for further details and raw data). In several other cases, technical errors with the software resulted in empty data files.

#### Contraction

For each twitch, the maximum absolute and relative force of contraction and the time taken to reach 100% of the maximum force of contraction were compared between DE50-MD and WT dogs (Fig. S2A). For tetanic traces, the time taken to reach 95% of the maximum absolute and relative force of contraction was compared (this metric proved more consistently reliable, as tetanic force often fluctuated throughout the 1 s run of pulses, precluding precise determination of 100%) (Fig. S2B). For this same reason, tetanic force was presented as the force produced at the end of the 1 s stimulation. To further characterise contraction for both twitch and tetanus stimulations, the time taken to reach 100% contraction was analysed in 10% intervals between 0-100% contraction to produce a time course for contraction.

#### Relaxation

For twitch and tetanic stimulations, time taken to relax (return to baseline) following peak contraction force was compared between genotypes Fig. S2A,B). The time taken to reach 95% relaxation was used as a proxy for complete relaxation rather than 100%, as in many cases, the force trace did not return precisely to baseline within the 0.8 s recording period. To further characterise relaxation for both twitch and tetanus stimulations, the time taken to reach 100% relaxation was analysed in 10% intervals between 0-100% relaxation to produce a time course for relaxation.

### ECD protocol

Following the twitch and tetanus protocol for both the TTJ flexors and extensors, the TTJ flexors were stimulated in an ECD protocol based on work by Tegeler et al. (2010) developed with the GRMD model. An initial isometric tetanic contraction was induced (50 Hz) and after 0.5 s of tetanic contraction, the computer induced rotation of the foot pedal by 29° over a 0.2 s period (∼0.7 muscle lengths per second), causing extension of the TTJ and thereby eccentric muscle stretch of approximately 15% during TTJ flexor muscle contraction (Fig. S3). This was repeated ten times with a 4 s gap between each stimulation. The sequence of ten stimulations was repeated twice, with a 4 min rest period between sequences (i.e. a total of 30 contractions per test) (Kornegay et al., 2011). The torque produced during the plateau phase of each tetanic contraction was quantified by the DMA software. Tetanic torque was measured at the end of the 0.5 s isometric (non-lengthening) contraction (Fig. S3). For each of the 30 tetanic contractions in the protocol, we divided the tetanic torque by the maximum tetanic torque produced during the protocol to calculate ECD. There were several reasons why data were excluded from analysis, the most common being due to the needle electrodes being dislodged during the protocol as a result of muscle movement during contraction. This resulted in abnormal force fluctuations, abnormally shaped tetani and/or missing datapoints (see ‘Eccentric contraction protocol data’ at https://doi.org/10.6084/m9.figshare.23501319 for more details and raw data). In other cases, datasets were excluded due to technical errors with the machine that resulted in missed datapoints or incorrect movement of the foot pedal.

### Fibre typing

#### Muscle sampling

Cranial tibial muscle samples were collected post-mortem (at 18 months of age) from four WT (WT-G2, WT J1, WT-K5 and WT-M2) and five DE50-MD (DE50-E4, DE50-H6, DE50-I1, DE50-I2 and DE50-T6) dogs. Muscles were bisected and the entire central portion was mounted in transverse orientation onto corks. CryoMbed mounting medium (Bright Instruments) was applied to the base of the piece of muscle to attach it to the cork (cryoMbed was not used to cover the whole muscle sample). The mounted sample was frozen in liquid nitrogen-cooled isopentane. Samples were stored at −80°C until use.

#### Immunohistochemistry

Cranial tibial muscle samples were transversely sectioned on a cryostat (Bright Instruments, OTF5000) at a thickness of 8 µm and the entire cross-section of the muscle was mounted onto glass slides (Superfrost Plus). Sections were air-dried for 30 min and then stored at −80°C until use (additional sections were collected in pre-chilled microcentrifuge tubes for RNA isolation, see below). Prior to staining, sections were removed from the −80°C freezer and placed at room temperature for 30 min, then fixed in 1:1 methanol:acetone at −20°C for 15 min. Slides were then washed over 5 min in three changes of PBS. Sections were incubated with rabbit collagen VI antibody (1:1000, Abcam, ab6588) for 60 min at room temperature. Slides were washed again over 5 min in three changes of PBS. Sections were then incubated with Alexa Fluor 647 goat anti-rabbit antibody (1:1000, Invitrogen, A32733) for 60 min at room temperature, before washing again in PBS. A Zenon labelling mixture (Invitrogen) was then prepared: per slide, 2 µl mouse anti-embryonic slow antibody (1:50, Millipore, MAB1628) was combined with 5 µl Alexa Fluor 350 (Invitrogen, Z25000), 5 µl mouse anti-embryonic myosin (1:20, Novocastra, NCL-MHCd) was combined with 5 µl Alexa Fluor 488 (Invitrogen, Z25002), and 0.1 µl mouse anti-myosin fast isoform (1:1000, Abcam, ab51263) was combined with 5 µl Alexa Fluor 594 (Invitrogen, Z25007). These individual antibody-Zenon labelling combinations were incubated for 5 min before addition of 5 µl Zenon blocking reagent (1:1 with Zenon label) for a further 5 min at room temperature. All antibodies were combined, PBS was added to make a final volume of 100 µl per slide, and the antibody mixture was applied to muscle sections for 60 min at room temperature, before washing over 5 min in three changes of PBS. 4% paraformaldehyde in PBS was then applied to each section for 15 min at room temperature, before washing in PBS. Glass coverslips were mounted with Prolong Gold Antifade Mountant (Invitrogen, 11539306) and imaged using an upright fluorescent microscope (Leica Microsystems, DM4000_Fluor). For each cranial tibial muscle imaged, five randomly selected 10× fields of view were imaged and analysed, and the mean results of these five images were used for further analysis.

#### Fibre type quantification

Following immunohistochemical staining for MHCs described above, fibre types were quantified using a semi-automated system using Fiji/ImageJ software and a recently developed protocol (https://www.protocols.io/private/2615235EA59311EA9EDB0A58A9FEAC2A). Briefly, images are imported into ImageJ using the bio-formats importer (plugins→bio-formats→bio-formats windowless importer) and converted to 8-bit four-channel stacks. Each stack is split into individual images of the four fluorescence channels used in the staining and imaging protocol above, and the collagen VI channel is inspected. Aberrant signal bleed-through is removed by subtracting the Alexa Fluor 594 channel image from the collagen VI-Alexa Fluor 647 channel using the ‘image calculator’ setting. This subtracted image is used to create a black and white mask that outlines each muscle fibre by thresholding from 1 to 255 (Image→Adjust→Threshold) and then generating a mask (Edit→Selection→Create mask). Each fibre is fully outlined for subsequent particle analysis by inverting the mask (Edit→Invert) followed by a binary watershed pass (Process→Binary→ Watershed). Fibres are then identified and numbered using the ‘analyse particles’ tool (settings: pixel units, 300-∞; circularity, 0.40-1.00; exclude on edges) and the resulting regions of interest (ROIs) are saved. This automated fibre detection protocol typically detects approximately 75% of fibres; consequently, some manual curation of fibres is required for complete and accurate fibre identification. This is achieved by overlaying the saved ROIs onto the original multichannel image. The ‘freehand selection’ tool is used to select incorrect ROIs and to manually draw correct fibre outlines. Downstream analysis of the original image and the corresponding set of ROIs is performed using the ImageJ macro ‘FibreClassifier.ijm’ available to download in the protocol (https://www.protocols.io/private/2615235EA59311EA9EDB0A58A9FEAC2A). The macro automatically measures mean fluorescence values within each ROI (muscle fibre) and uses the result to assign each ROI to specific fibre types, of which seven can be detected: fast, slow, fast/slow hybrid, pure regenerative (embryonic MHC only), fast/regenerative hybrid, slow/regenerative hybrid and fast/slow/regenerative hybrid. For the purposes of this study, pure regenerative, fast/regenerative hybrid, slow/regenerative hybrid and fast/slow/regenerative hybrid fibres were grouped together to give a single value for regenerative fibres during analysis of the results. Threshold parameters are adjusted within each experiment. For each image analysed, the macro produces three thresholding plots showing the fluorescence values for each ROI: a red-blue plot, a green-blue plot and a green/blue-green/red plot. The resulting fibre type assignation can be seen via a composite image. Thresholds for fibre type assignation should be adjusted based on analysis of ROI clustering on the three plot windows and comparison of the resulting fibre type assignation with the original image. Thresholds can be adjusted using the ‘Parameters’ setting window. Once the user is satisfied with the adjustments, the ‘accept and draw’ option is selected to produce a list of the ROI fluorescence values and fibre type assignment. These parameters can then be saved and used to analyse the images in batches.

#### RT-qPCR

RNA was isolated and RT-qPCR conducted as previously described (Riddell et al., 2022), from the same samples used for histological analysis (as described above): following sectioning and mounting for histology, additional unmounted serial cryosections (20-40, approximately 100 mg of tissue) were collected directly into a pre-chilled 1.5 ml Eppendorf tube. RNA was isolated from the frozen tissue using TRIzol (Invitrogen, 15596026) according to the manufacturer's instructions, with the addition of a 1:1 chloroform extraction step and using a modified precipitation protocol [isopropanol (one volume), 3 M sodium acetate pH 5.5 (0.1 volume) and 10 µg/ml glycogen]. RNA was quantified using a spectrophotometer (NanoDrop 1000, Thermo Fisher Scientific) and mRNA was reverse transcribed using 1600 ng total RNA per 20 µl reaction, with oligo(dT) and random nonamer priming (RTnanoscript2, Primerdesign). cDNAs were then diluted 1:20 to a final concentration of 4 ng/µl (assuming a 1:1 conversion).

Quantitative PCRs were performed in duplicate or triplicate using PrecisionPLUS PCR master mix with SYBR green (Primerdesign) and 2 µl of the 4 ng/µl cDNA described above for a total of ∼8 ng cDNA per reaction. Reactions were performed using a CFX384 thermal cycler (Bio-Rad), beginning with a denaturation step of 95°C for 3 min, followed by 40 cycles of the following parameters: 95°C for 15 s, 60°C for 20 s and 72°C for 20 s. Melt curves were performed for all reactions: 60 to 95°C, in 0.5°C increments every 5 s. Three reference genes with stable muscle expression in both WT and DE50-MD dogs (Hildyard et al., 2018b) were used to normalise the data: ribosomal protein L13a (*RPL13A*), hypoxanthine phosphoribosyltransferase 1 (*HPRT1*) and succinate dehydrogenase (*SDHA*), with primers from the geNorm *Canis familiaris* set (Primerdesign). Primer sequences for these three reference genes are proprietary; however, we provide anchor nucleotides and context length (Table S8). HPRT1 primers were designed to span introns, precluding amplification of genomic sequence under the PCR conditions used, and a single sharp amplicon peak was seen for all samples. For the primer pairs for *RPL13A* and *SDHA* that did not span introns, no-reverse-transcriptase controls were tested to establish levels of genomic DNA within the samples; the results confirmed that gDNA did not significantly contribute to Cq values (data available in Hildyard et al., 2018b). Primers to MHCs were designed using Primer3 (https://primer3.ut.ee/) and target unique sequence regions of each isoform (and span introns) (Table S9). The relative expression of each gene of interest was quantified relative to the three reference genes *HPRT1*, *RPL13A* and *SDHA*, and analysed in log form. Additionally, raw Cq values were compared to estimate the relative expression of each MHC isoform (see ‘RT-qPCR fibre typing relative values’ at https://doi.org/10.6084/m9.figshare.23501319).

### Statistical analysis

Linear mixed models were used to compare results between different aged animals, between WT and DE50-MD genotypes, and between different ages within each genotype for: twitch and tetanus maximum force; time to 100% contraction or 95% relaxation; twitch:tetanus torque ratio; and ECD. Post hoc analysis was performed using Holm–Šídák correction for multiple comparisons. Linear regression analysis was used to assess the relationship between maximum absolute tetanic force during the ECD protocol and the tetanic torque remaining following 30 ECs (percentage of maximum torque during the protocol), and between the age and tetanic torque remaining following 30 ECs. The differences were considered statistically significantly when *P*<0.05. Fibre type composition of the cranial tibial muscle determined by immunohistochemistry and RT-qPCR was analysed by multiple two-tailed unpaired *t*-tests corrected for multiple comparisons by the Holm–Šídák method. Linear mixed modelling and post hoc analyses were performed using IBM SPSS Statistics v28. Graphs were produced and multiple two-tailed unpaired *t*-tests with Holm–Šídák correction were performed using GraphPad Prism 8.0. Sample size calculations were performed using GLIMMPSE online software (Kreidler et al., 2013), using a two-way repeated measures ANOVA model and a desired power of 0.8.

## Supplementary Material

10.1242/dmm.050395_sup1 Supplementary information
