# Supplementary material for: Longitudinal assessment of skeletal muscle functional mechanics in the DE50-MD dog model of Duchenne muscular dystrophy
Source: Dis Model Mech. 2023 Dec 20;16(12):dmm050395. doi: 10.1242/dmm.050395 (PMC10753191; doi:10.1242/dmm.050395)
Supplement: Supplementary information [file dmm-16-050395-s1.pdf]

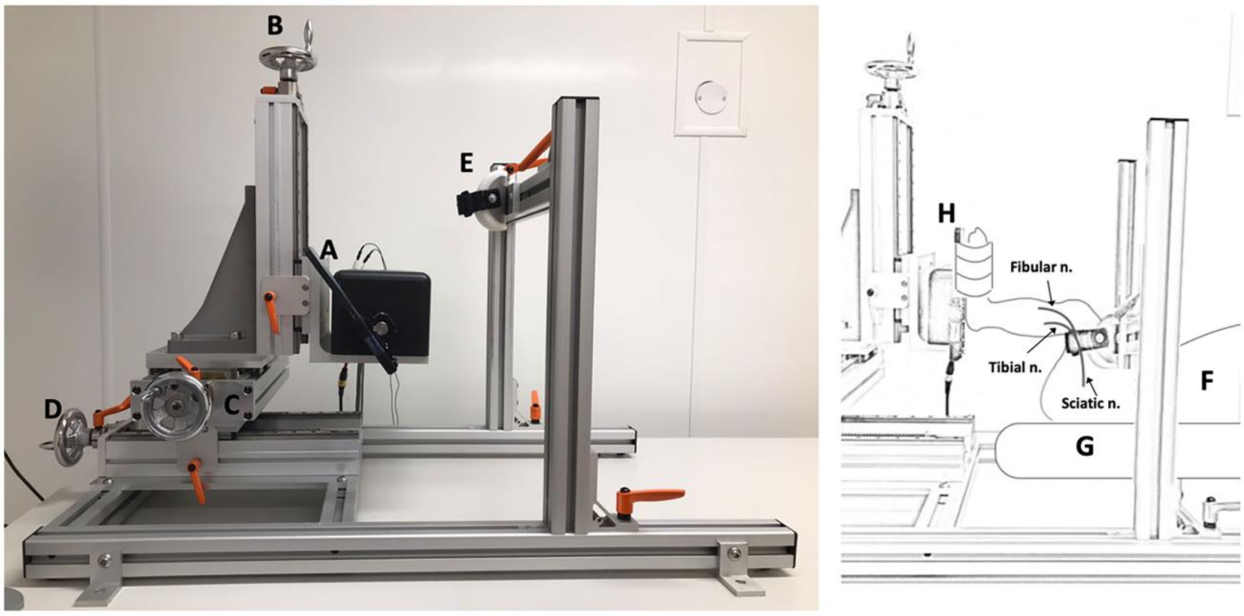

Fig. S1. Aurora Scientific large animal frame and muscle torque control system. (A) Force transducing foot-pedal connected to a Dual-Mode Lever system (#310C-FP). The foot-pedal is mounted on a tri-axial system allowing adjustments to the y-axis (B), x-axis (C) and z-axis (D). (E) Foam pad on a height-adjustable bar, for resting of the cranial aspect of the thigh. Under general anaesthesia, test animals (F) were placed on a blanket (G) in dorsal recumbency on the table with the tibiotarsal (TTJ), stifle and coxo-femoral joints positioned at 90° angles. The foot (tarsus) was taped to a force-transducing foot-pedal (H). The 2 branches of the sciatic nerve, the tibial nerve supplying the TTJ extensors and the fibular nerve supplying the TTJ flexors, were stimulated individually by needle electrodes placed percutaneously, and the resulting force exerted on the foot-pedal was recorded.

Disease Models & Mechanisms • Supplementary information

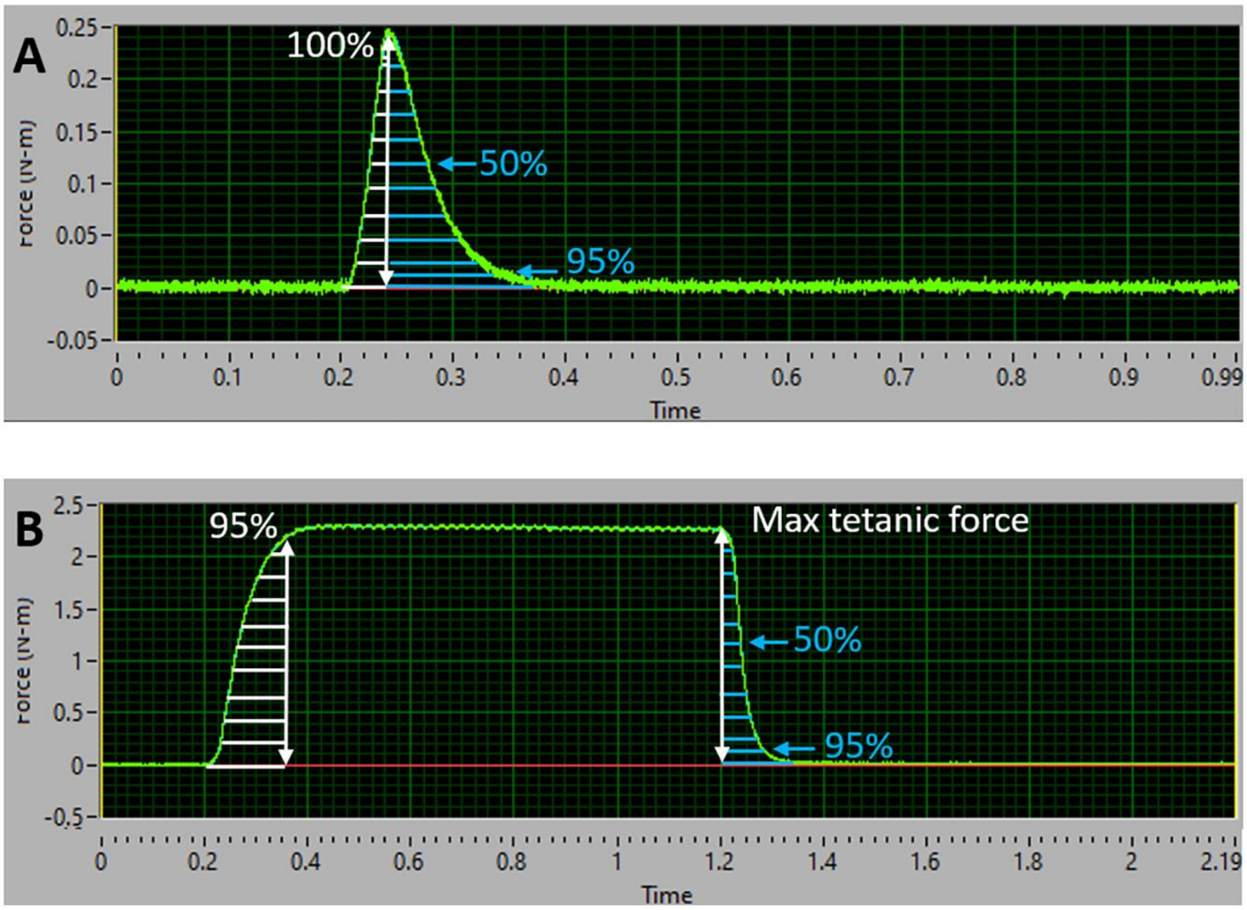

Fig. S2. Muscle contraction and relaxation parameters analysed for each twitch and tetanus. Annotated example traces from Aurora Scientific Dynamic Muscle Analysis Software. Time in seconds is displayed on the x-axis and torque (labelled "Force") in Nm on the y-axis. Parameters relating to muscle contraction are labelled in white, those relating to relaxation are labelled in blue. The green line shows torque (Nm) exerted onto the foot-pedal following nerve stimulation and resultant muscle contraction. The red line shows baseline torque. A) Parameters quantified for each twitch trace were the maximum force of contraction (labelled 100%), time taken to reach 100% of maximum contraction, and time taken to reach 95% relaxation following the peak of maximum contraction. In addition, the time taken to reach peak contraction and relaxation was analysed in 10% increments. B) Parameters quantified for each tetanus trace were the force of contraction at the end of the 1s stimulation period (max tetanic force), time taken to reach 95% of maximum contraction following start of electrode stimulation (at 0.2s), and time taken to reach 95% relaxation following the end of stimulation (at 1.2s). The time taken to reach peak contraction and relaxation was also analysed in 10% increments.

Disease Models & Mechanisms • Supplementary information

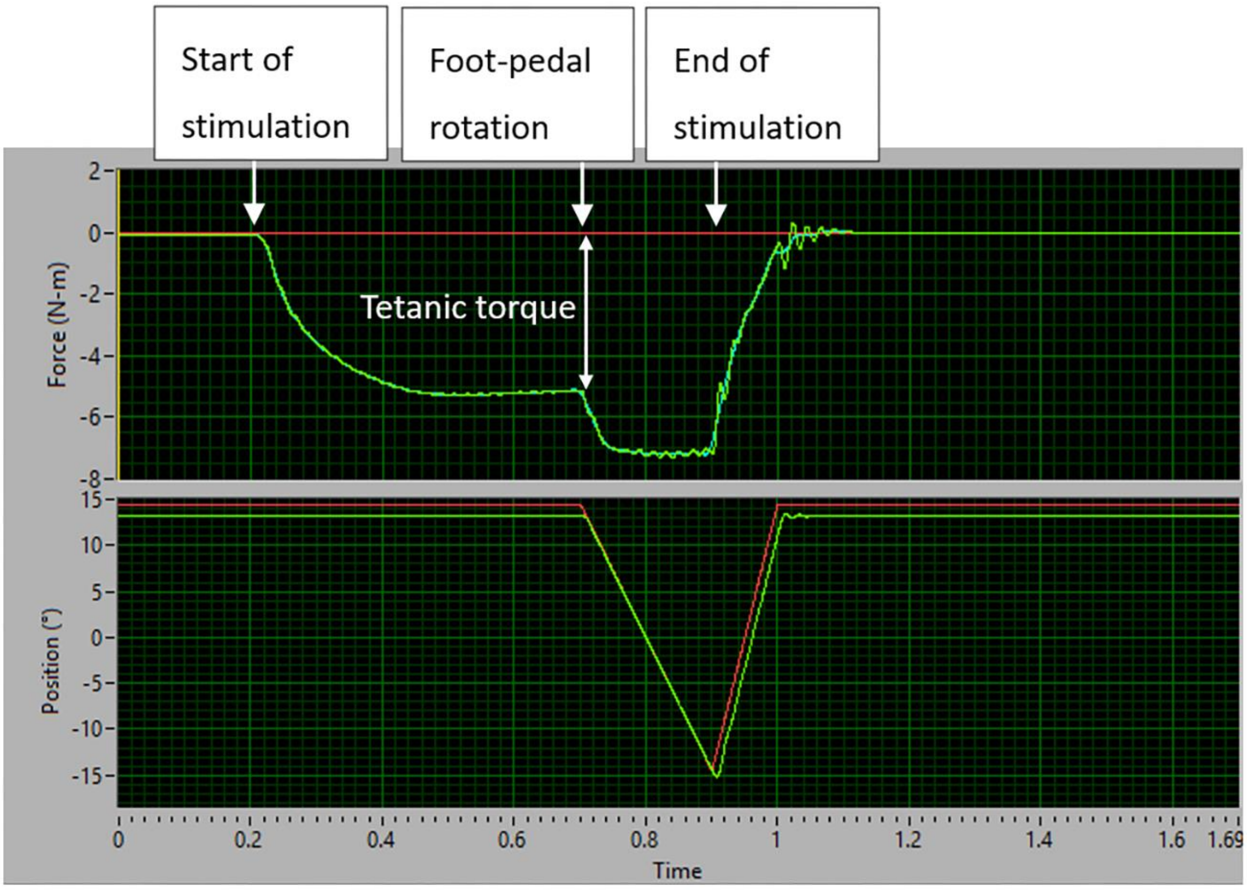

Fig. S3. Example of eccentric contraction decrement protocol torque trace. Time in seconds (s) is displayed on the x-axis. Torque (Nm) is displayed on the y-axis of the upper trace (labelled "Force") and position of the foot-pedal is displayed on the y-axis of the lower trace, revealing the 29° rotation during the tetanus. Electrode stimulation of the fibular nerve is initiated at 0.2s and continues for 0.7s. Stimulation induces a tetanus in the muscles that flex the tibiotarsal joint (TTJ). The green line shows torque (Nm) exerted onto the foot-pedal following nerve stimulation and resultant muscle contraction. The red line shows baseline torque. After 0.5s of tetanic contraction, the foot-pedal rotates, extending the TTJ joint and lengthening the contracting muscles. The lower half of the graph shows the position of the foot-pedal: the red line shows the position that the computer has been programmed to move the foot-pedal to, and the green line shows the actual position of the foot-pedal during the contraction. This acts a quality control measure, to confirm that the foot-pedal is moving as expected.

Disease Models & Mechanisms • Supplementary information

Table S1. Data summary for TTJ flexors and extensors twitch and tetanus absolute force. Mean (N), standard deviation, minimum and maximum (N), and N-number of animals for WT and DE50-MD genotypes. P-value shows whether the difference in mean result between WT and DE50-MD was significant based on linear mixed model analysis corrected for multiple comparisons by the Holm-Šídák method.

			WT					DE50-MD					WT vs DE50-MD
			Mean	SD	Min	Max	N	Mean	SD	Min	Max	N	p-value
Twitch and tetanus - absolute force (N)	TTJ flexors twitch absolute	3	0.47	0.22	0.23	0.84	6	0.20	0.12	0.06	0.33	6	0.69
	TTJ flexors twitch absolute	6	1.32	1.22	0.25	4.02	10	0.28	0.30	0.09	1.11	10	0.07
	TTJ flexors twitch absolute	9	2.66	2.48	0.09	6.91	9	0.95	0.67	0.33	2.13	9	0.01
	TTJ flexors twitch absolute	12	1.55	0.68	0.72	2.71	11	1.11	1.01	0.17	3.46	10	0.50
	TTJ flexors twitch absolute	15	2.00	0.74	1.09	2.95	10	0.84	0.56	0.28	1.92	8	0.05
	TTJ flexors twitch absolute	18	3.49	2.35	0.86	8.05	11	1.51	0.90	0.47	2.90	9	0.0007
	TTJ flexors twitch absolute	All ages	2.03	1.78	0.09	8.05	57	0.85	0.80	0.06	3.46	52	0.0004
	TTJ extensors twitch absolute	3	1.52	0.71	0.84	2.59	6	1.08	0.71	0.50	2.32	6	0.78
	TTJ extensors twitch absolute	6	5.05	2.73	2.01	11.04	10	2.81	1.37	0.66	5.25	10	0.09
	TTJ extensors twitch absolute	9	5.58	2.41	3.25	9.85	10	4.14	2.90	0.24	8.59	9	0.31
	TTJ extensors twitch absolute	12	7.17	3.93	0.51	14.20	11	3.35	1.67	0.61	6.96	10	0.004
	TTJ extensors twitch absolute	15	5.81	3.11	1.51	13.20	10	4.03	2.54	1.71	9.22	7	0.24
	TTJ extensors twitch absolute	18	9.64	5.07	3.35	19.01	11	4.52	2.77	1.40	10.05	9	0.0002
	TTJ extensors twitch absolute	All ages	6.18	4.00	0.51	19.01	58	3.42	2.31	0.24	10.05	51	0.002
	TTJ flexors tetanus absolute	3	4.79	3.22	1.96	10.26	6	2.08	1.27	0.62	3.57	6	0.47
	TTJ flexors tetanus absolute	6	13.52	7.39	3.40	25.58	10	2.94	2.07	0.78	7.64	10	0.0004
	TTJ flexors tetanus absolute	9	16.35	9.67	6.24	35.67	10	6.74	3.55	3.01	14.21	9	0.002
	TTJ flexors tetanus absolute	12	13.63	5.49	5.68	23.32	11	7.43	5.63	1.97	15.56	10	0.03
	TTJ flexors tetanus absolute	15	16.96	7.66	9.29	32.24	10	9.07	5.40	1.92	16.26	8	0.01
	TTJ flexors tetanus absolute	18	22.11	10.09	11.10	42.68	11	11.40	4.71	4.97	20.13	9	0.0004
	TTJ flexors tetanus absolute	All ages	15.35	8.89	1.96	42.68	58	6.77	5.11	0.62	20.13	52	<0.0001
	TTJ extensors tetanus absolute	3	10.57	5.60	4.45	21.11	6	7.06	4.19	2.95	13.55	6	0.69
	TTJ extensors tetanus absolute	6	23.55	11.81	11.62	45.60	10	18.94	9.28	3.05	32.49	10	0.43
	TTJ extensors tetanus absolute	9	23.35	7.99	10.05	35.51	9	20.66	10.85	4.30	34.74	9	0.76
	TTJ extensors tetanus absolute	12	27.43	14.67	4.20	57.29	11	20.41	8.79	5.71	37.58	10	0.19
	TTJ extensors tetanus absolute	15	29.57	13.83	7.30	55.67	10	20.30	10.53	9.95	39.54	7	0.15
	TTJ extensors tetanus absolute	18	32.18	16.46	9.09	67.67	10	24.05	10.39	8.08	40.51	9	0.13
	TTJ extensors tetanus absolute	All ages	25.51	13.69	4.20	67.67	56	19.22	10.17	2.95	40.51	51	0.11

Disease Models & Mechanisms • Supplementary information

Table S2. Data summary for TTJ flexors and extensors twitch and tetanus relative force. Mean (N/kg), standard deviation, minimum and maximum (N/kg), and N-number of animals for WT and DE50-MD genotypes. P-value shows whether the difference in mean result between WT and DE50-MD was significant based on linear mixed model analysis corrected for multiple comparisons by the Holm-Šídák method.

			WT					DE50-MD					WT vs DE50-MD
		Age	Mean	SD	Min	Max	N	Mean	SD	Min	Max	N	p-value
Twitch and tetanus - relative force (N/kg)	TTJ flexors twitch relative	3	0.09	0.04	0.05	0.14	6	0.05	0.04	0.02	0.10	6	0.56
	TTJ flexors twitch relative	6	0.14	0.13	0.03	0.44	10	0.04	0.06	0.01	0.20	10	0.08
	TTJ flexors twitch relative	9	0.26	0.24	0.01	0.66	9	0.12	0.08	0.04	0.26	9	0.01
	TTJ flexors twitch relative	12	0.14	0.07	0.06	0.30	11	0.13	0.10	0.02	0.31	10	0.89
	TTJ flexors twitch relative	15	0.18	0.07	0.10	0.27	10	0.10	0.07	0.03	0.22	8	0.16
	TTJ flexors twitch relative	18	0.30	0.19	0.07	0.64	11	0.17	0.09	0.06	0.33	9	0.01
	TTJ flexors twitch relative	All ages	0.19	0.16	0.01	0.66	57	0.10	0.09	0.01	0.33	52	0.002
	TTJ extensors twitch relative	3	0.30	0.11	0.19	0.45	6	0.29	0.16	0.14	0.53	6	0.91
	TTJ extensors twitch relative	6	0.55	0.30	0.27	1.26	10	0.42	0.25	0.10	0.95	10	0.30
	TTJ extensors twitch relative	9	0.54	0.23	0.32	0.97	10	0.50	0.31	0.03	1.00	9	0.77
	TTJ extensors twitch relative	12	0.63	0.32	0.05	1.18	11	0.41	0.23	0.07	0.99	10	0.10
	TTJ extensors twitch relative	15	0.52	0.33	0.16	1.35	10	0.44	0.18	0.20	0.76	7	0.58
	TTJ extensors twitch relative	18	0.85	0.47	0.35	1.84	11	0.50	0.29	0.20	1.15	9	0.01
	TTJ extensors twitch relative	All ages	0.59	0.35	0.05	1.84	58	0.43	0.25	0.03	1.15	51	0.05
	TTJ flexors tetanus relative	3	0.92	0.53	0.46	1.78	6	0.61	0.41	0.14	1.11	6	0.41
	TTJ flexors tetanus relative	6	1.49	0.81	0.36	2.81	10	0.43	0.37	0.12	1.38	10	0.0004
	TTJ flexors tetanus relative	9	1.58	0.95	0.61	3.50	10	0.85	0.46	0.33	1.71	9	0.02
	TTJ flexors tetanus relative	12	1.22	0.47	0.47	1.94	11	0.89	0.65	0.21	1.75	10	0.24
	TTJ flexors tetanus relative	15	1.52	0.73	0.79	2.97	10	1.04	0.58	0.20	1.90	8	0.12
	TTJ flexors tetanus relative	18	1.93	0.82	1.04	3.41	11	1.28	0.42	0.60	1.84	9	0.03
	TTJ flexors tetanus relative	All ages	1.49	0.77	0.36	3.50	58	0.86	0.55	0.12	1.90	52	<0.0001
	TTJ extensors tetanus relative	3	2.06	0.89	1.01	3.65	6	1.91	1.09	1.02	3.88	6	0.82
	TTJ extensors tetanus relative	6	2.62	1.34	1.22	5.21	10	2.77	1.45	0.47	4.51	10	0.77
	TTJ extensors tetanus relative	9	2.25	0.77	0.95	3.35	9	2.51	1.20	0.52	4.04	9	0.63
	TTJ extensors tetanus relative	12	2.39	1.17	0.41	4.75	11	2.50	1.20	0.70	5.33	10	0.88
	TTJ extensors tetanus relative	15	2.66	1.41	0.75	5.71	10	2.24	0.88	1.28	3.27	7	0.61
	TTJ extensors tetanus relative	18	2.84	1.55	0.96	6.54	10	2.68	1.03	1.14	4.63	9	0.75
	TTJ extensors tetanus relative	All ages	2.50	1.22	0.41	6.54	56	2.48	1.15	0.47	5.33	51	0.95

Table S3. Data summary for TTJ flexors and extensors twitch-tetanus torque ratio. Mean (AU), standard deviation, minimum and maximum (AU), and N-number of animals for WT and DE50-MD genotypes. P-value shows whether the difference in mean result between WT and DE50-MD was significant based on linear mixed model analysis corrected for multiple comparisons by the Holm-Šídák method.

			WT					DE50-MD					WT vs DE50-MD
		Age	Mean	SD	Min	Max	N	Mean	SD	Min	Max	N	p-value
Twitch-tetanus torque ratio (AU)	TTJ flexors twitch tetanus ratio	3	0.11	0.03	0.08	0.16	6	0.11	0.04	0.05	0.17	6	0.96
	TTJ flexors twitch tetanus ratio	6	0.10	0.05	0.01	0.21	10	0.11	0.06	0.02	0.26	10	0.85
	TTJ flexors twitch tetanus ratio	9	0.14	0.07	0.01	0.22	9	0.13	0.05	0.07	0.22	9	0.71
	TTJ flexors twitch tetanus ratio	12	0.12	0.05	0.06	0.26	11	0.14	0.06	0.07	0.24	10	0.44
	TTJ flexors twitch tetanus ratio	15	0.14	0.07	0.03	0.24	10	0.10	0.04	0.06	0.15	8	0.07
	TTJ flexors twitch tetanus ratio	18	0.15	0.05	0.06	0.21	11	0.13	0.05	0.04	0.21	9	0.23
	TTJ flexors twitch tetanus ratio	All ages	0.13	0.06	0.01	0.26	57	0.12	0.05	0.02	0.26	52	0.43
	TTJ extensors twitch tetanus ratio	3	0.15	0.04	0.11	0.22	6	0.15	0.04	0.11	0.21	6	0.99
	TTJ extensors twitch tetanus ratio	6	0.22	0.05	0.15	0.31	10	0.16	0.06	0.08	0.27	10	0.04
	TTJ extensors twitch tetanus ratio	9	0.24	0.08	0.14	0.38	9	0.18	0.06	0.06	0.25	9	0.02
	TTJ extensors twitch tetanus ratio	12	0.26	0.10	0.12	0.42	11	0.16	0.03	0.11	0.22	10	0.0002
	TTJ extensors twitch tetanus ratio	15	0.20	0.06	0.11	0.28	10	0.18	0.03	0.15	0.23	6	0.52
	TTJ extensors twitch tetanus ratio	18	0.30	0.06	0.21	0.40	10	0.18	0.05	0.10	0.25	9	<0.0001
	TTJ extensors twitch tetanus ratio	All ages	0.24	0.08	0.11	0.42	56	0.17	0.05	0.06	0.27	50	0.0001

Disease Models & Mechanisms • Supplementary information

Table S4. Data summary for TTJ flexors and extensors time to contraction. Mean (s), standard deviation, minimum and maximum (s), and N-number of animals for WT and DE50-MD genotypes. P-value shows whether the difference in mean result between WT and DE50-MD was significant based on linear mixed model analysis corrected for multiple comparisons by the Holm-Šídák method.

			WT					DE50-MD					WT vs DE50-MD
		Age	Mean	SD	Min	Max	N	Mean	SD	Min	Max	N	p-value
Time to contraction (s)	TTJ flexors twitch 100% contraction	3	0.041	0.002	0.039	0.043	6	0.049	0.006	0.041	0.057	5	0.004
	TTJ flexors twitch 100% contraction	6	0.045	0.004	0.040	0.055	10	0.053	0.006	0.044	0.059	9	<0.0001
	TTJ flexors twitch 100% contraction	9	0.047	0.006	0.039	0.059	9	0.044	0.002	0.042	0.049	9	0.23
	TTJ flexors twitch 100% contraction	12	0.047	0.004	0.042	0.055	11	0.043	0.005	0.038	0.054	10	0.01
	TTJ flexors twitch 100% contraction	15	0.048	0.005	0.043	0.059	10	0.044	0.005	0.041	0.056	8	0.13
	TTJ flexors twitch 100% contraction	18	0.049	0.003	0.045	0.053	11	0.044	0.005	0.041	0.056	9	0.01
	TTJ flexors twitch 100% contraction	All ages	0.047	0.005	0.039	0.059	57	0.046	0.006	0.038	0.059	50	1.00
	TTJ extensors twitch 100% contraction	3	0.060	0.013	0.038	0.076	6	0.060	0.003	0.056	0.065	6	0.97
	TTJ extensors twitch 100% contraction	6	0.091	0.022	0.054	0.127	10	0.068	0.008	0.058	0.084	10	0.0007
	TTJ extensors twitch 100% contraction	9	0.096	0.020	0.070	0.131	10	0.067	0.014	0.053	0.091	9	<0.0001
	TTJ extensors twitch 100% contraction	12	0.086	0.019	0.055	0.115	11	0.060	0.005	0.054	0.069	10	<0.0001
	TTJ extensors twitch 100% contraction	15	0.086	0.016	0.064	0.104	10	0.063	0.007	0.055	0.072	7	0.0014
	TTJ extensors twitch 100% contraction	18	0.098	0.018	0.068	0.124	11	0.058	0.004	0.053	0.066	9	<0.0001
	TTJ extensors twitch 100% contraction	All ages	0.088	0.021	0.038	0.131	58	0.063	0.009	0.053	0.091	51	<0.0001
	TTJ flexors tetanus 95% contraction	3	0.167	0.050	0.111	0.253	6	0.345	0.096	0.254	0.502	6	0.02
	TTJ flexors tetanus 95% contraction	6	0.252	0.162	0.128	0.564	10	0.314	0.130	0.139	0.531	10	0.27
	TTJ flexors tetanus 95% contraction	9	0.288	0.177	0.124	0.623	10	0.289	0.139	0.160	0.541	9	0.51
	TTJ flexors tetanus 95% contraction	12	0.194	0.103	0.106	0.479	11	0.222	0.135	0.109	0.582	10	0.60
	TTJ flexors tetanus 95% contraction	15	0.289	0.117	0.122	0.494	10	0.221	0.065	0.125	0.318	8	0.26
	TTJ flexors tetanus 95% contraction	18	0.192	0.083	0.129	0.425	11	0.244	0.156	0.127	0.609	9	0.36
	TTJ flexors tetanus 95% contraction	All ages	0.233	0.129	0.106	0.623	58	0.269	0.129	0.109	0.609	52	0.05
	TTJ extensors tetanus 95% contraction	3	0.326	0.074	0.204	0.416	6	0.354	0.093	0.213	0.491	6	0.70
	TTJ extensors tetanus 95% contraction	6	0.298	0.105	0.198	0.496	10	0.398	0.149	0.206	0.638	10	0.08
	TTJ extensors tetanus 95% contraction	9	0.317	0.104	0.177	0.487	9	0.367	0.147	0.241	0.612	9	0.40
	TTJ extensors tetanus 95% contraction	12	0.225	0.106	0.106	0.494	11	0.361	0.124	0.196	0.581	10	0.01
	TTJ extensors tetanus 95% contraction	15	0.356	0.181	0.154	0.700	10	0.408	0.140	0.239	0.626	7	0.40
	TTJ extensors tetanus 95% contraction	18	0.208	0.075	0.116	0.367	10	0.326	0.136	0.185	0.636	9	0.04
	TTJ extensors tetanus 95% contraction	All ages	0.284	0.124	0.106	0.700	56	0.369	0.131	0.185	0.638	51	0.001

Table S5. Data summary for TTJ flexors and extensors time to relaxation. Mean (s), standard deviation, minimum and maximum (s), and N-number of animals for WT and DE50-MD genotypes. P-value shows whether the difference in mean result between WT and DE50-MD was significant based on linear mixed model analysis corrected for multiple comparisons by the Holm-Šídák method.

			WT					DE50-MD					WT vs DE50-MD
		Age	Mean	SD	Min	Max	N	Mean	SD	Min	Max	N	p-value
Time to relaxation (s)	TTJ flexors twitch 95% relaxation	3	0.130	0.081	0.082	0.293	6	0.515	0.259	0.214	0.755	6	0.0002
	TTJ flexors twitch 95% relaxation	6	0.098	0.030	0.059	0.153	10	0.340	0.304	0.061	0.755	10	0.002
	TTJ flexors twitch 95% relaxation	9	0.104	0.019	0.077	0.132	9	0.313	0.272	0.101	0.756	9	0.002
	TTJ flexors twitch 95% relaxation	12	0.106	0.026	0.078	0.153	11	0.326	0.294	0.054	0.756	10	0.001
	TTJ flexors twitch 95% relaxation	15	0.114	0.021	0.086	0.163	10	0.207	0.228	0.095	0.759	8	0.07
	TTJ flexors twitch 95% relaxation	18	0.115	0.023	0.089	0.170	11	0.255	0.284	0.095	0.757	9	0.06
	TTJ flexors twitch 95% relaxation	All ages	0.110	0.034	0.059	0.293	57	0.318	0.277	0.054	0.759	52	0.001
	TTJ extensors twitch 95% relaxation	3	0.218	0.060	0.142	0.309	6	0.396	0.267	0.180	0.740	6	0.002
	TTJ extensors twitch 95% relaxation	6	0.204	0.034	0.156	0.280	10	0.397	0.237	0.183	0.736	10	0.0001
	TTJ extensors twitch 95% relaxation	9	0.242	0.051	0.177	0.337	10	0.259	0.062	0.140	0.336	9	0.50
	TTJ extensors twitch 95% relaxation	12	0.225	0.036	0.175	0.296	11	0.276	0.034	0.224	0.319	10	0.18
	TTJ extensors twitch 95% relaxation	15	0.228	0.033	0.167	0.280	10	0.259	0.036	0.219	0.330	7	0.43
	TTJ extensors twitch 95% relaxation	18	0.220	0.038	0.174	0.301	11	0.266	0.052	0.192	0.349	9	0.21
	TTJ extensors twitch 95% relaxation	All ages	0.223	0.041	0.142	0.337	58	0.307	0.150	0.140	0.740	51	0.02
	TTJ flexors tetanus 95% relaxation	3	0.104	0.020	0.081	0.141	6	0.385	0.326	0.107	0.800	6	0.05
	TTJ flexors tetanus 95% relaxation	6	0.095	0.012	0.066	0.106	10	0.486	0.332	0.123	0.800	10	0.0001
	TTJ flexors tetanus 95% relaxation	9	0.098	0.013	0.075	0.118	9	0.349	0.338	0.101	0.800	9	0.002
	TTJ flexors tetanus 95% relaxation	12	0.090	0.010	0.079	0.111	11	0.244	0.294	0.064	0.800	10	0.02
	TTJ flexors tetanus 95% relaxation	15	0.094	0.006	0.086	0.107	10	0.195	0.245	0.078	0.800	8	0.17
	TTJ flexors tetanus 95% relaxation	18	0.095	0.009	0.080	0.108	11	0.172	0.236	0.065	0.800	9	0.21
	TTJ flexors tetanus 95% relaxation	All ages	0.095	0.012	0.066	0.141	57	0.305	0.305	0.064	0.800	52	0.01
	TTJ extensors tetanus 95% relaxation	3	0.211	0.063	0.153	0.325	6	0.285	0.254	0.140	0.800	6	0.29
	TTJ extensors tetanus 95% relaxation	6	0.201	0.026	0.166	0.256	10	0.378	0.294	0.134	0.800	10	0.001
	TTJ extensors tetanus 95% relaxation	9	0.192	0.023	0.159	0.224	9	0.210	0.116	0.152	0.517	9	0.69
	TTJ extensors tetanus 95% relaxation	12	0.181	0.019	0.155	0.224	11	0.180	0.063	0.132	0.350	10	0.99
	TTJ extensors tetanus 95% relaxation	15	0.178	0.013	0.162	0.202	10	0.167	0.009	0.158	0.181	7	0.85
	TTJ extensors tetanus 95% relaxation	18	0.190	0.008	0.175	0.201	10	0.179	0.066	0.116	0.338	9	0.87
	TTJ extensors tetanus 95% relaxation	All ages	0.191	0.028	0.153	0.325	56	0.235	0.178	0.116	0.800	51	0.08

Disease Models & Mechanisms • Supplementary information

Table S6. Data summary for TTJ flexors eccentric contraction induced force decline. Eccentric contraction (ECC) force is presented as force of the last tetanic contraction in the protocol as a percentage of the maximum tetanic force produced during the protocol. Mean (%), standard deviation, minimum and maximum (%), and N-number of animals for WT and DE50-MD genotypes. P-value shows whether the difference in mean result between WT and DE50-MD was significant based on linear mixed model analysis corrected for multiple comparisons by the Holm-Šídák method.

			WT					DE50-MD					WT vs DE50-MD
		Age	Mean	SD	Min	Max	N	Mean	SD	Min	Max	N	p-value
ECC force (last as a % of max)	ECC force	3	79.7	12.3	61.4	100.0	6	69.8	12.8	54.8	92.7	6	0.45
	ECC force	6	81.3	16.1	60.8	100.0	8	45.0	17.5	17.6	83.0	10	<0.0001
	ECC force	9	83.2	11.5	67.6	100.0	8	32.8	16.9	13.4	53.3	9	<0.0001
	ECC force	12	79.4	10.4	59.0	94.7	11	24.0	12.7	6.5	45.8	9	<0.0001
	ECC force	15	89.9	6.1	82.3	98.3	9	24.0	17.0	1.9	60.1	9	<0.0001
	ECC force	18	82.2	12.9	54.9	98.4	12	18.4	6.1	9.0	29.1	8	<0.0001
	ECC force	All ages	82.7	11.8	54.9	100.0	54	34.2	21.2	1.9	92.7	51	<0.0001

Disease Models & Mechanisms • Supplementary information

Table S7. Cranial tibial (TTJ flexor) muscle fibre type quantification data summary for 18-month old dogs. Mean (%), standard deviation (SD) and range (%) for WT (N=4) and DE50-MD (N=5) genotypes for immunohistochemical (IHC) and RT-qPCR based methods of fibre type quantification. P-value indicates whether the difference in mean result between WT and DE50-MD was significant based on multiple T-test analysis corrected for multiple comparisons by the Holm-Šídák method.

		WT			DE50-MD			WT vs DE50-MD
		Mean (%)	SD	Range (%)	Mean (%)	SD	Range (%)	p-value
IHC	Type I fibres	40.55	9.35	32-53	19.64	5.27	15-27	0.01
	Type IIA/X fibres	58.12	8.94	46-66	69.10	4.13	65-75	0.06
	Type I/II hybrid fibres	1.09	0.48	0.7-1.7	4.27	1.34	2.3-5.7	0.01
	Regenerating fibres	0.25	0.11	0.1-0.4	6.99	4.93	1.6-13.7	0.06
RT-qPCR Log relative quantity	MHC1beta (type I fibres)	-0.34	0.34	-0.7-0.1	-1.10	0.42	-1.7--0.7	0.06
	MHC2A (type IIA fibres)	-0.39	0.34	-0.6-0.1	-0.67	0.37	-1.2--0.3	0.47
	MHC2X (type IIX fibres)	-0.39	0.35	-0.7-0.1	-0.50	0.47	-1.3--0.3	0.69
	MHC2B (type IIB fibres)	-1.01	0.65	-1.9--0.3	-0.06	0.08	-0.2-0.0	0.05
	MHCemb (regen fibres)	-3.01	0.21	-3.2--2.8	0.00	0.39	-0.5-0.6	0.00002
	MHCneo (regen fibres)	-1.91	0.82	-3.1--1.3	-0.17	0.23	-0.5-0.0	0.01
RT-qPCR Relative expression	MHC1beta (type I fibres)	15.94	6.32	10-25	2.99	1.09	1.8-4.5	0.01
	MHC2A (type IIA fibres)	47.31	4.08	41-51	27.31	7.25	20-36	0.01
	MHC2X (type IIX fibres)	35.85	7.01	26-39	32.38	14.24	11-50	0.67
	MHC2B (type IIB fibres)	0.02	0.03	0.002-0.07	0.12	0.06	0.06-0.2	0.07
	MHCemb (regen fibres)	0.005	0.003	0.002-0.007	10.37	16.49	1.6-40	0.45
	MHCneo (regen fibres)	0.87	0.91	0.05-2.2	26.83	5.52	21-35	0.00002
RT-qPCR Relative expression Excluding regenerating MHCs	MHC1beta (type I fibres)	16.09	6.37	10-25	4.83	1.11	3.7-6.1	0.02
	MHC2A (type IIA fibres)	47.70	3.71	42-51	45.40	11.26	29-60	0.71
	MHC2X (type IIX fibres)	36.19	7.22	26-43	49.54	12.25	34-67	0.26
	MHC2B (type IIB fibres)	0.02	0.03	0.002-0.08	0.24	0.22	0.08-0.6	0.02

Disease Models & Mechanisms • Supplementary information

Table S8. Primer specifications for RT-qPCR reference genes

Primer (accession number)	Anchor nucleotide	Context length
HPRT1 (NM_001003357)	131	100
RPL13A (XM_003432726)	407	164
SDHA (XM_535807)	967	80

Table S9. Primer specifications for RT-qPCR myosin heavy chain (MHC) transcripts. For each transcript the transcript name, accession number, both forward (Fwd) and reverse (Rev) primer orientations and the primer sequence are provided.

Primer (Accession number)	Fwd/Rev	Sequence
MHC Slow1beta (XM_038672843)	Fwd	CTGATGCACCTGTCGAGAAG
MHC Slow1beta (XM_038672843)	Rev	TCACCCCTGGAGACTTTGTC
MHC 2A (XM_038664213)	Fwd	TCAAGGGGAGATCAGTGTGG
MHC 2A (XM_038664213)	Rev	TCTGGAGGTAGGCTGCTTTG
MHC 2B (NM_001076794)	Fwd	CAGAGTGGTGGAGTCAATGC
MHC 2B (NM_001076794)	Rev	CAATATTCCTTGCGTGTTTCTAAGA
MHC 2X (XM_038664035)	Fwd	TCTCACTGATCCACTACGCG
MHC 2X (XM_038664035)	Rev	CTTTCTTGCCACCACCAGCC
MHC embryonic (XM_038664165)	Fwd	GACGCGGTCTGTCAAGGG
MHC embryonic (XM_038664165)	Rev	TGCTGAGCTTTGCGGAATTT
MHC neonatal (XM_038664076)	Fwd	ATCAACGACCTGACAGCTCA
MHC neonatal (XM_038664076)	Rev	TCTTCCTCCAGCTGACGTTT

Disease Models & Mechanisms • Supplementary information
